# A Comprehensive Literature Review of Total Hip Arthroplasty (THA): Part 1—Biomaterials

**DOI:** 10.3390/jfb16050179

**Published:** 2025-05-14

**Authors:** Chiara Morano, Salvatore Garofalo, Paolo Bertuccio, Agata Sposato, Irene Zappone, Leonardo Pagnotta

**Affiliations:** Department of Mechanical, Energy and Management Engineering, University of Calabria, Via P. Bucci 44C, 87036 Rende, Italy; salvatore.garofalo@unical.it (S.G.);

**Keywords:** total hip arthroplasty (THA), total hip replacement (THR), bearing surfaces, biomaterials, material selection

## Abstract

The rapid advancement of materials science has revolutionized total hip arthroplasty (THA), a critical orthopedic procedure aimed at restoring mobility and improving patient quality of life. This review investigates the evolution of biomaterials used in THA, analyzing their mechanical, biological, and chemical properties. The study outlines the transition from early natural materials to modern metals, polymers, and ceramics, highlighting their benefits and limitations in clinical applications. Particular emphasis is placed on the development of advanced materials such as highly cross-linked polyethylene (HXLPE), zirconia-toughened alumina (ZTA), and tantalum alloys (Ta), which demonstrate enhanced biocompatibility, wear resistance, and longevity. By examining emerging trends, including bioactive coatings and nanotechnology, this paper aims to provide a comprehensive understanding of current challenges and future directions in material selection for hip prostheses, ultimately aiming to minimize annual revision rates and improve long-term outcomes.

## 1. Introduction

**Figure 1:**
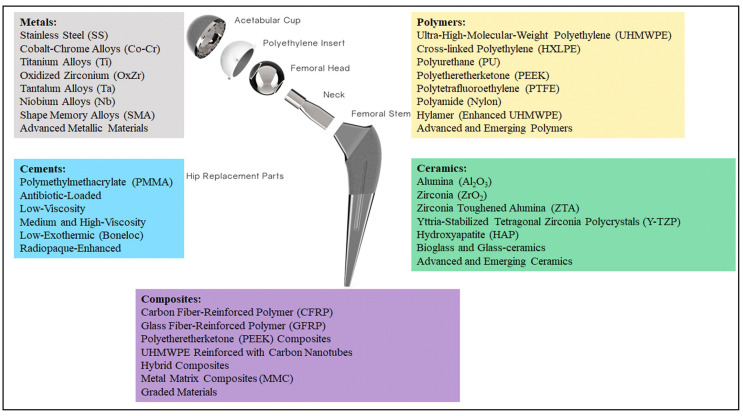
Biomaterials for THA.

Total hip arthroplasty (THA) is a surgical procedure that involves the substitution of a damaged bone for a prosthetic component. This procedure is complex, and it is one of the most widespread and effective orthopedic surgeries [1]. The surgical procedure is performed in several stages: (i) First of all, the damaged femoral head is removed; (ii) after that, a canal is created in the femur proximal medullary region; (iii) finally, an artificial prosthesis is inserted into the canal [2]. The new prosthesis is composed of two different components, as reported in Figure 1: a stem and an acetabular head, which in turn is realized by a femoral head, a liner, and an outer shell.

In recent years, the number of hip replacement surgeries performed annually worldwide has increased [1,3]. This occurrence is related to different phenomena, e.g., the aging population, the increasingly reliable surgical techniques, and the greater availability of logistical, instrumental, and human resources. This leads to the need to develop solutions for longer lifespans, thus reducing the risk of premature failure, i.e., new surgery requests.

The reliability of the THA procedure depends on different factors. Among them, one of the most influential factors is material selection. Indeed, material issues are one of the main causes of implant failure. This is related to different occurrences, e.g., mechanical wear, infection, or material degradation [4,5]. Due to this, the right material selection is crucial since it could directly affect the durability and the reliability of the implant itself.

Historically, the materials used in hip prostheses have seen significant evolution. At the beginning, i.e., in the late 19th and early 20th centuries, natural materials, e.g., wood, ivory, and glass, were employed. However, even if these materials appeared to be innovative at the time, different issues related to their poor mechanical properties or susceptibility to breakage and infections reveal that this kind of material was inadequate [6]. As surgical techniques advanced, early synthetic materials such as rubber and Pyrex glass were experimented with; however, these also proved insufficient for hip joint replacement demands.

By the mid-20th century, the focus had shifted to metals, with stainless steel (SS) and cobalt-chrome (Co-Cr) alloys becoming commonly used in hip prostheses. While these materials offered improved strength and durability, they often failed due to corrosion and wear [6,7]. This led to the adoption of titanium (Ti) alloys, known for their superior strength and biocompatibility, and Ultra-High-Molecular-Weight PolyEthylene (UHMWPE), which provided enhanced wear resistance, marking significant progress in THA [8].

The literature specifically dedicated to biomaterials used in the construction of components for THA is extensive. Over the past decade, numerous review papers focusing on biomaterials for THA have been published [9,10,11,12,13], reflecting the significant interest among researchers. Moreover, different research papers discuss the influence of biomaterial selection on THA [14,15]. Different studies have been carried out for each of the five fundamental components of a modern total hip prosthesis: the stem; the cement used for cemented prostheses; the ball (femoral head), which may or may not be a single piece with the stem; the liner placed between the femoral head and the acetabular cup that can be fixed to the pelvis; and finally the acetabular cup. However, the literature lacks a comprehensive approach for comparing different materials for each prosthesis component and providing an instrument for proper material selection. With this aim, two different review papers were developed, with a focus on material properties (Part 1) and material selection criteria (Part 2).

Regarding material properties, the discussion of materials is divided into four main categories: metals, ceramics, polymers, and composites (see Figure 1). Moreover, a further category can be distinguished considering the cements that are very valuable for part mating. These biomaterials were developed in order to enhance the overall living conditions of the patients. Moreover, they could also help improve implant reliability, avoiding the need for repeated surgeries. Different materials have been employed in order to obtain the best combination of biocompatibility and mechanical properties. The aim of this paper is to provide information on the most commonly used materials for manufacturing each component of the implant. To this end, all recommendations shared in the literature have been considered [16,17,18,19,20,21,22,23,24,25]. Finally, it should be noted that this paper is the first part of a broader study: while Part 1 provides an in-depth comparative analysis of the most commonly used materials and biomaterials in THA—including their clinical performance, associated complication rates, and revision outcomes—Part 2 focuses specifically on the selection criteria and decision-making methods for choosing appropriate materials.

## 2. Literature Analysis Method

**Figure 2:**
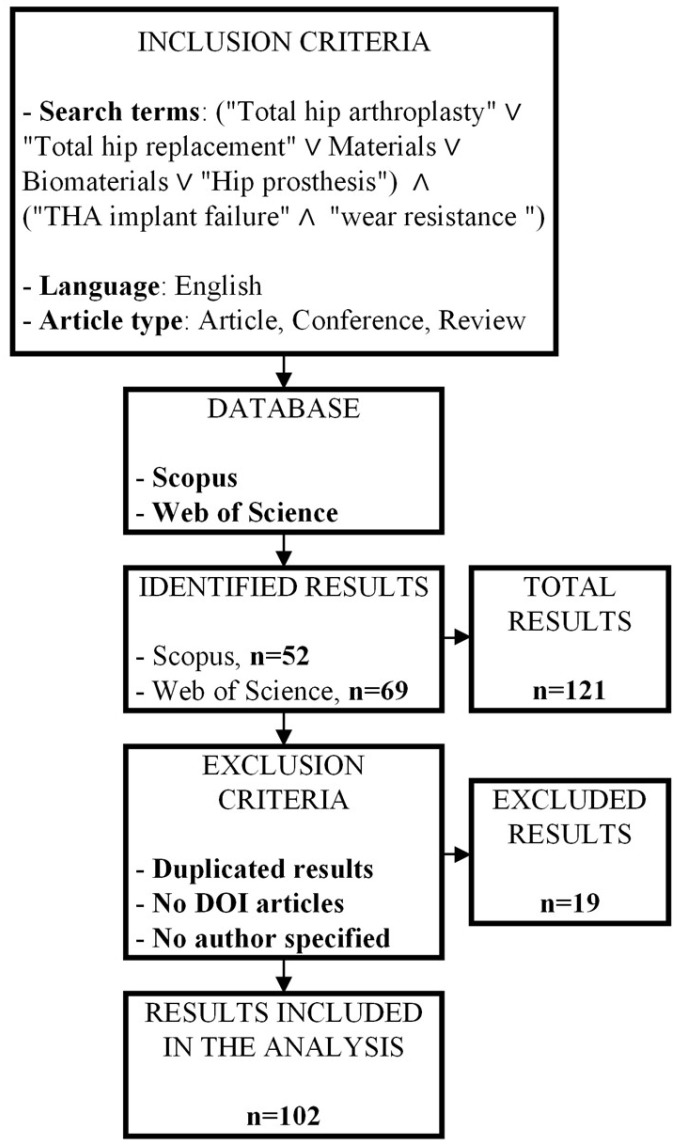
Literature analysis scheme.

The literature analysis was conducted following a methodical approach. The overall process is summarized in Figure 2.

First, the inclusion criteria were defined based on the expected outcomes. In particular, the selected keywords included “Total hip arthroplasty”, “Materials”, “Biomaterials”, and “Hip prosthesis”. Moreover, since one of the goals was to evaluate the performance of different materials for THA applications, the criteria “THA implant failure” and “wear resistance” were also included.

Finally, the results were limited to articles, conference papers, and reviews, and only documents written in English were considered.

The research was carried out using different databases, namely Scopus and Web of Science. Duplicate results were excluded, as well as all articles that could not be reliably identified, e.g., those lacking author names or DOI information.

Overall, 19 articles were excluded from the analysis. The screening process led to the identification of 102 articles, which were analyzed in depth and are discussed in the following sections.

## 3. Metals

**Figure 3:**
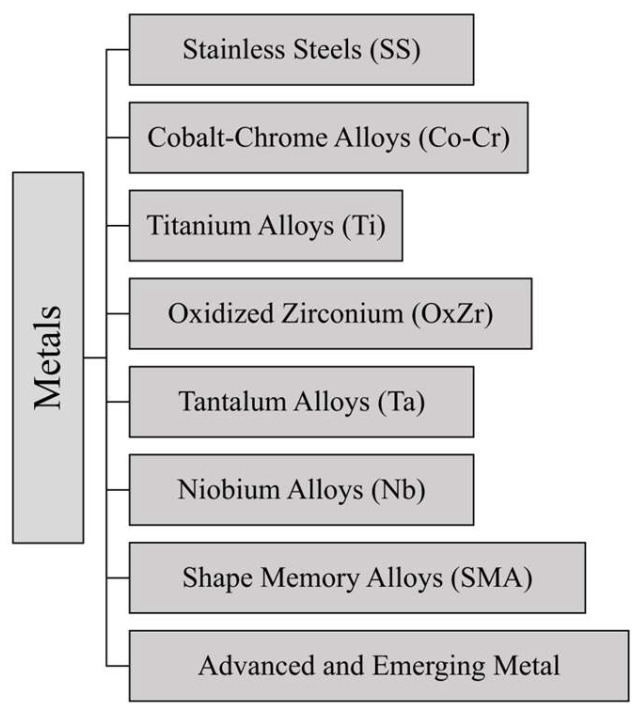
Main metallic materials employed for THA.

Metallic materials have played a critical role in the development of THA since they are able to guarantee requirements demanded by human body operating conditions, i.e., adequate mechanical strength and reliability. Metals, like SS, Co-Cr alloys, and Ti alloys, have been employed in THA surgery from early times, thanks to their biocompatibility as well as their mechanical properties [23,26]. In fact, metals exhibit good resistance to fatigue (i.e., repetitive stresses of daily activities), good wear behavior, and a low release of harmful ions into the body [26]. A summary of the main metals employed for THA surgery is reported in Figure 3.

In recent years, different advanced alloys with improved resistance to corrosion and wear, i.e., critical factors in the long-term success of hip implants, have been developed for THA applications [12,14]. The choice of metal is influenced by its ability to integrate with bone and reduce adverse reactions, ensuring the implant’s reliability and durability.

As a result, ongoing research continues to refine these materials, focusing on enhancing their performance and extending the lifespan of hip implants, ultimately contributing to better patient outcomes [14,23,26]. The details of the metallic materials used in THA are provided below, followed by Table 1, which reports their main mechanical properties and clinical metrics. Additionally, Table S1 summarizes the key benefits, drawbacks, and applications of metallic materials.

### 3.1. Stainless Steel (SS)

SS has been widely employed in THA thanks to its specific properties, i.e., corrosion resistance, mechanical strength, and biocompatibility. Among different types of SS, the most widespread are the AISI 316 and 316 L, thanks to their excellent corrosion resistance.

The composition of 316L SS includes different elements, i.e., Fe, Cr (17–19%), Ni (13–15%), Mo (2–3%), Mn (up to 2%), C (<0.03%), and other elements, that could be included in order to improve corrosion resistance and reduce metal ion toxicity [23].

SS was first used in medicine in 1926. In 1938, Philip Wiles performed the first total hip replacement (THR) with SS, utilizing a specially made implant secured with screws and bolts [24]. However, these early hip replacements had significant issues with wear and loosening due to high friction and the poor quality of SS employed at the time. In order to address this problem, SS was gradually replaced by cobalt-chrome–molybdenum alloys in the so-called second-generation Metal-on-Metal (MoM) implants [27,28]. Due to its ability to withstand corrosion in biological conditions, which is crucial for avoiding the release of metal ions that may result in unfavorable bodily responses, SS has nevertheless been utilized for parts like femoral stems and femoral heads [9,10].

When paired with an HXLPE liner in primary hip prosthesis, SS femoral heads have a lower revision rate than ion-implanted Co-Cr heads (LFIT) according to recent research. This implies that SS can provide better performance in terms of endurance and revision rate even if it is a less costly and technically less sophisticated material than treated Co-Cr [29].

316L SS was given a novel Ti-6Al-4V-2ZrC coating to improve its qualities even more. It has been demonstrated that this coating could increase the hardness, wear resistance, and tribocorrosion resistance of 316L SS. Thanks to that, this material has become a good choice for hip replacement parts like the femoral head and acetabular cup. Moreover, the coating improves biocompatibility and lowers the dangers associated with long-term usage of uncoated 316L SS by lowering the production of metal ions such as Ni, Fe, and Cr [30].

Another type of SS used in cemented femoral stems is high-nitrogen SS, which is especially well liked in Australia and the UK. This material is a recommended option for particular applications because of its excellent mechanical qualities and corrosion resistance [26].

Although SS is widely used, there are some drawbacks related to the material employment. First of all, SS tends to deteriorate over time as a result of corrosion and fatigue, particularly in physiological conditions and under dynamic loads. Metal ions, including Fe, Cr, and Ni, may be released as a result, which could impair biocompatibility and cause issues such as implant loosening [9,12,14,31]. Additionally, metallosis, pseudotumors, and periprosthetic bone loss can result from the discharge of metal ions, which is especially troublesome for patients who are metal-sensitive [27]. SS has a higher elastic modulus than other materials used in THA, such as Ti alloys and ceramics. This can result in “stress shielding” and shorten the implant’s lifespan [14,25,29].

Because of these problems, Ti and its alloys, which have superior corrosion resistance and biocompatibility, have supplanted SS as a biomedical material for hip replacement [12]. However, SS remains a popular choice in many parts of the world, especially for low-cost applications and for elderly or less active patients, where mechanical strength is more critical than long-term durability [25,29]. SS is more cost-effective than materials like Ti, but it sacrifices biocompatibility and long-term corrosion resistance in the process [9].

Despite technological improvements that have led to the use of more advanced materials in current hip prostheses, its continuous usage shows its significance in certain settings.

### 3.2. Cobalt-Chrome Alloys (Co-Cr)

Co-Cr alloys are among the most widely used materials in THA surgery due to their excellent mechanical properties and corrosion resistance. These alloys are primarily composed of cobalt (Co), with additions of chromium (Cr), molybdenum (Mo), and small amounts of other elements such as nickel (Ni) and iron (Fe), which contribute to their strength and biocompatibility. The typical composition of a Co-Cr alloy used in THA is the remaining Co, Cr 27–30%, Mo 5–7%, Ni max 1%, Fe max 0.75%, and C max 0.35%, with the possible presence of other elements such as manganese (Mn) and tungsten (W) [9,20,31].

Co-Cr alloys were first used for hip prostheses fabrication in the 1930s, with significant improvements in the 1960s due to reduced wear rates and enhanced corrosion resistance. Their application expanded significantly during the 20th century, with global adoption in orthopedic surgery, particularly for young and active patients, due to their durability [22].

In the context of THA, Co-Cr alloys are mainly used for the femoral head and metal articulation surfaces, where their wear resistance and low friction properties are critical [22,31]. However, despite the numerous advantages, they also present some drawbacks, such as the release of metal ions and the potential for adverse reactions in the body, including the formation of pseudotumors and the necrosis of soft tissues [19,32,33]. Mihalko et al. [27] discuss in detail the issues related to corrosion and ion release from different metallic alloys, emphasizing the importance of surface coatings and ceramics as alternatives to improve biocompatibility and reduce adverse reactions. Santavirta et al. [28] highlight the performance of Co-Cr alloys in MoM bearing couples, stressing the need for precise geometries to minimize wear and impingement risks, while also emphasizing the effectiveness of modern self-polishing techniques to maintain low wear rates.

Aherwar et al. [31] emphasize the superiority of cobalt-based alloys over other metallic materials, such as Ti and SS, due to their excellent corrosion resistance, fatigue resistance, and high mechanical properties, making them ideal for MoM bearing couples and components such as femoral stems. Joshi et al. [14] confirm that the Co-Ni-Cr-Mo (Wrought) alloy emerges as the most suitable for hip prostheses, thanks to its high mechanical strength, minimal stress and deformation, and high safety factor, outperforming other alloys such as Ti and its variants. Babazadeh et al. [29] compared SS femoral heads with ion-implanted Co-Cr heads and found that the former had a lower revision rate, particularly with 32 mm diameter heads. This suggests that, despite the potential benefits of ion implantation, SS may be preferable due to its lower cost. McTighe et al. [26] discuss the manufacturing processes of Co-Cr alloys, highlighting how forging and casting affect the mechanical properties and corrosion resistance of prosthetic components, emphasizing the importance of material selection based on specific in vivo operating conditions. Finally, Hamidi et al. [34] point out that while Co-Cr alloys have excellent mechanical properties, they may pose biocompatibility issues due to ion release and their high stiffness compared to natural bone, which can lead to bone overload and implant loosening.

Co-Cr alloys are widely used in THA worldwide, with significant prevalence over other materials such as Ti alloys and ceramics. Compared to Ti, Co-Cr alloys offer greater wear resistance but are less biocompatible, while compared to ceramics, Co-Cr alloys have higher fracture resistance but higher wear rates [31,34].

### 3.3. Titanium Alloys (Ti)

Thanks to their superior mechanical qualities and biocompatibility, Ti alloys and, in particular, Ti-6Al-4V, could be considered as an industry standard for THA. These alloys, which were first introduced in the 1970s, are mostly made of Ti (90%), Al (6%), and V (4%). They provide the perfect ratio of mechanical strength to light weight. Notwithstanding these benefits, their low wear resistance—particularly in femoral heads—has restricted their usage in some particular applications [9,10].

The first application of Ti for THA was in the 1950s. However, a widespread application of this material did not occur until the 1970s. Ti alloys are particularly valued for femoral stems and acetabular cups due to their resistance to corrosion and compatibility with human tissues [24].

Different methods allow the development of two new Ti alloys, i.e., Ti-6Al-7Nb and Ti-13Nb-13Zr, for THA surgery. These materials were developed to overcome the limitations of traditional Ti alloys by improving mechanical strength and biocompatibility and reducing the toxicity hazards associated with aluminum and vanadium. Additionally, their better fatigue resistance and lower elastic modulus, which is more in line with human bone, lead to a reduction in stress shielding, a situation that could eventually compromise the stability of implants [35,36].

Through surface coatings, contemporary technologies have further increased the efficacy of Ti prostheses. For instance, by improving osteoblast adhesion and stimulating bone formation surrounding the implant, coatings containing Hydroxyapatite (HAP) and other bioactive compounds greatly enhance osseointegration. Long-term stability depends on these gains, especially for patients who are young and active [37].

The elastic modulus of Ti alloys, which is comparable to that of human bone, is one of its best qualities. Additionally, research has indicated that Ti femoral components, which are utilized in cementless hip replacement, exhibit outstanding long-term performance in young patients, with follow-ups lasting anywhere from five to eleven years [38]. Wear resistance is still a major problem despite these benefits [10]. Furthermore, when combining various metallic alloys, such as Ti and Co-Cr, within implants, McTighe et al. drew attention to the possibility of galvanic corrosion [26].

For many THA applications worldwide, Ti alloys remain the material of choice, particularly in cementless constructions. Most patients, especially those who need longer-lasting implants, find them perfect due to their outstanding biomechanical qualities, remarkable corrosion resistance, and capacity to integrate well with bone tissue [14,38].

### 3.4. Oxidized Zirconium (OxZr)

In orthopedic implants, OxZr has grown in popularity, especially for THA. This material is made by heat-treating zirconium alloy, which preserves the metal core while forming a strong, wear-resistant ceramic surface. The end product is a material that is perfect for parts like femoral heads in hip implants because it combines the hardness and fracture resistance of metals with the wear resistance and biocompatibility of ceramics [17,24].

A zirconium (Zr) base alloyed with additional elements, like Niobium (Nb), is the usual component of OxZr. This alloy’s metallic core is preserved while its surface oxidizes to form a hard, ceramic-like coating [10]. Compared to more conventional materials like Co-Cr alloys, this special structure drastically reduces wear rates, which results in less wear of the polyethylene liners in hip replacements—a crucial component of these implants’ lifetime [19].

In the early 2000s, OxZr was initially used in orthopedic surgery; in 2002, its first clinical application was documented. The necessity for a material that could provide the wear resistance of ceramics without the brittleness that goes along with it fueled its development. The difficulties previously encountered by both ceramic and metal materials employed in THA were addressed by the coupling of a ceramic surface with a long-lasting metallic core [19,24].

The main application of OxZr in THA is in the femoral heads. It has been a key development in prolonging the life of hip implants due to its capacity to drastically reduce the wear of polyethylene liners, especially in younger, more active patients, where the wear resistance of the implant is more demanding [17,39]. Additionally, it is a great option for people who are sensitive to metals due to their lower roughness values and biocompatibility when compared to Co-Cr alloys [10].

The key advantages of OxZr include its superior wear resistance, which reduces the generation of wear particles and thus decreases the risk of osteolysis. It also features better biocompatibility and a lower coefficient of friction, hence resulting in lower rates of metal ion hypersensitivity reactions. In addition, the toughness that is provided by the metallic core makes sure that the fractures, which are a big issue with ceramic-only components, are not seen [17,19].

However, there are some drawbacks. The production of OxZr is expensive, and the process of manufacturing it is quite complicated. Also, while OxZr has been shown to produce less wear, the data on its performance over the long term when compared with traditional materials are still being collected, and OxZr is not easily available as compared to the other materials [39].

OxZr is used all over the world, and the demand for it is especially high in the United States and Europe. This implant is used where there is a need to minimize the wear because the patient is relatively young, and the implants may be required to last for more than a decade. This material is also used in patients with metal allergies, as it has low ion release due to its ceramic surface [19].

When compared to Co-Cr alloys, OxZr is highly effective in reducing the wear rates of polyethylene liners, which is very important for the success of hip implants. It also provides improved biocompatibility, reducing the occurrence of hypersensitivity reactions to metal ions. When compared to traditional ceramics, OxZr offers the added advantage of toughness, reducing the risk of catastrophic failure due to fractures [40,41].

### 3.5. Tantalum Alloys (Ta)

The transition metal Ta is renowned for its remarkable chemical stability, biocompatibility, and corrosion resistance. It is the perfect material for orthopedic prosthetics because of these qualities, particularly THA [42,43]. Ta’s capacity to create a protective oxide layer on its surface, which protects it from the corrosive effects of physiological fluids, is one of its main advantages. This helps to extend the life of implants [44].

Ta is usually pure when used in prostheses; however, it can be alloyed with other metals, including Nb, to enhance certain mechanical qualities. Because of its three-dimensional structure, porous Ta, which is produced using sophisticated sintering techniques, promotes superior osseointegration. This porous material, called “Trabecular Metal”, has a porosity of 75% to 85% and is intended to resemble cancellous bone [42,44].

Ta has been used medicinally since the 1940s, but its usage in orthopedic prosthetics—specifically in THA—did not gain traction until the 1990s. Significant increases in implant stability and longevity have resulted from the material’s growing use in the manufacture of hip prosthesis components due to its special qualities [43,44].

Ta is frequently utilized in the production of femoral stems, acetabular inserts for THA, and acetabular cups. Because Ta is porous, it can integrate strongly with the surrounding bone, increasing implant durability and lowering the risk of migration. In revision procedures, where bone loss and other complicated situations call for treatments that offer excellent initial stability, Ta is very useful. Monoblock acetabular components constructed of Ta, which have a high-density polyethylene liner directly molded into the porous component, are one example of recent developments [37,44].

Ta’s great biocompatibility, which lowers the possibility of negative reactions and promotes improved bone integration, is one of its main benefits. Furthermore, because the protective oxide layer shields it from deterioration in hostile biological settings, its corrosion resistance guarantees long-term durability within the body. Clinical research has shown that Ta acetabular cups had 100% survival rates even 12 years after surgery, demonstrating the material’s superior osseointegration properties [45].

Ta does, however, have several disadvantages. Its accessibility in areas with limited resources is restricted by the complexity and expense of its production. Furthermore, while its high stiffness helps stabilize implants, it may be a drawback in situations where more flexibility is needed [44]. Ta’s high coefficient of friction might also make it more difficult to position implants optimally during surgery, especially when carrying out primary arthroplasty.

Ta, one of the most cutting-edge materials for hip prostheses, is mostly utilized in North America and Europe. Ta’s exorbitant price, however, prevents it from being widely used in other regions of the world. Ta may be more appropriate for revision procedures according to recent registry studies that have questioned its efficacy in primary hip surgery when compared to alternative materials [43,44,46].

Ta has better osseointegration and corrosion resistance than other materials like Ti and Co-Cr. However, Co-Cr is chosen for its resistance to wear, while Ti is frequently picked for its reduced weight. Ta may not provide the same advantages in main procedures when exact implant placement is crucial, even though it is helpful in complicated situations like revision surgeries [43].

### 3.6. Niobium Alloys (Nb)

In the realm of orthopedic implants, particularly in THA, Nb alloys have gained prominence, especially when coupled with Ti and zirconium (Zr). The lifespan of hip replacements depends on these alloys’ remarkable mechanical and biocompatible qualities, which include increased fatigue strength and corrosion resistance. The necessity to provide exceptional resistance to wear and corrosion within the human body while minimizing the discharge of hazardous ions—a typical problem with other metallic materials—led to the creation of Nb alloys [20,47].

The most often mentioned alloy is Ti-13Nb-13Zr, which is composed of a Ti matrix with roughly 13% Nb and 13% Zirconium (Zr). It has been demonstrated that this particular composition improves the mechanical performance and biocompatibility of implants, especially concerning corrosion and wear resistance. The requirement for hypoallergenic materials led to the addition of Nb to Ti alloys since, in contrast to certain other metals, Nb does not cause negative bodily reactions [19,48].

In the past, Nb-based alloys were well known as substitutes for conventional materials such as Co-Cr alloys, which were linked to increased levels of ion release and possible negative tissue reactions. An important development in the early 2000s was the incorporation of Nb into Ti alloys, which combined mechanical durability with biocompatibility [19,47].

Excellent biocompatibility and hypoallergenic qualities and higher mechanical performance in terms of wear and fatigue resistance are only a few benefits of Nb alloys [49,50]. The costlier production of these advanced alloys than traditional materials, however, and the requirement for further long-term clinical data to validate their benefits across a range of patient populations are the main disadvantages [47].

Globally, sophisticated orthopedic procedures mostly employ Nb alloys in North America and Europe.

Nb alloys are more appropriate for younger, more active patients because they have superior mechanical qualities and increased resilience to wear and fatigue when compared to SS [9].

### 3.7. Shape Memory Alloys (SMAs)

SMAs, particularly Nickel–Titanium (NiTi) alloys like Nitinol, are recognized for their unique ability to return to a pre-defined shape after being plastically deformed, thanks to the “shape memory effect” and superelasticity. These properties are highly advantageous in medical applications, especially in THA, where SMAs can enhance initial implant stability and reduce stress on bone tissue [18].

Introduced in the 1990s, SMAs have leveraged their fatigue resistance, biocompatibility, and mechanical adaptability to become particularly suitable for components such as femoral stems and fixation plates in THA. Their ability to dynamically adapt to movement significantly reduces the risk of “stress shielding”, a phenomenon that can compromise the longevity of the implant [51]. SMAs like Nitinol are currently extensively used in biomedical applications due to their excellent biocompatibility, high corrosion resistance, and low modulus of elasticity, which closely mimics the mechanical behavior of bone [52].

Moreover, SMAs are used to enhance the initial stability of uncemented femoral stems. Their ability to expand and conform to the shape of the medullary canal improves initial contact and implant stability [51]. However, challenges related to micromotion at the bone–implant interface persist, and further studies are necessary to optimize the use of these materials [51].

The ability of NiTi alloys to simulate the biomechanical properties of natural tissues, combined with their shape memory effect and pseudoelasticity, makes them ideal for applications in hip prostheses [52]. However, challenges remain, particularly concerning the release of nickel ions, which can cause allergic reactions and other biocompatibility issues [22,52]. Ongoing research focuses on developing alternative alloys, such as those based on Nb, to offer improved biocompatibility while maintaining the desirable properties of SMAs [22]. Their potential for use in advanced manufacturing techniques, like additive manufacturing, opens new possibilities for customized and optimized implants [27]. However, issues related to biocompatibility and corrosion resistance still need to be addressed, with suggestions for improving SMAs through new coatings or material combinations [52].

Innovative uses of SMAs include their application in modular acetabular insert revision systems. These systems utilize the unique properties of SMAs to generate sufficient forces to facilitate the removal of ceramic inserts without damaging the surrounding metal components, thereby improving the safety and effectiveness of revision procedures [53].

### 3.8. Advanced Metallic Materials

Advanced metallic materials such as Ti alloys Ti-25Nb-2Mo-4Sn, Ti-15Zr-4Nb-4Ta, and Ti-35Nb-7Zr-5Ta are important advancements in the field of THA. These materials are specifically made to reduce the problems that are typically associated with metallic implants and improve biomechanical compatibility with the human body. These alloys reduce stress shielding, a problem where stronger implants transfer less load to the surrounding bone, potentially resulting in bone resorption and implant failure, by decreasing the elastic modulus, which better matches the mechanical characteristics of human bone [9].

Coatings like Diamond-Like Carbon (DLC) and Titanium Nitride (TiN) are essential for enhancing these implants’ longevity. These coatings greatly increase the implants’ lifespan by preventing wear on the metal surfaces underneath. They also aid in lowering metal ion release, a typical problem with metallic implants that can result in negative biological reactions such as metal hypersensitivity and inflammation [10].

The creation of metal–ceramic composites and bioactive coatings is a recent development. To improve osseointegration—the process by which bone cells adhere and develop onto the surface of the implant—bioactive coatings, such as HAP and Bioglass, are applied to Ti alloys. For the implant to remain stable in the bone over time, this improvement is essential. In order to lessen wear and increase the implant’s overall lifetime, metal–ceramic composites combine the mechanical strength of metals with the biocompatibility of ceramics [12,21].

These cutting-edge materials have historically been created to overcome the drawbacks of previous, more conventional materials used in THA. A constant attempt to enhance the functionality and security of hip implants is shown in the ongoing research into metallic coatings like TiN and DLC, bioactive coatings, and metal–ceramic composites. Even though these developments represent a major step forward, rather than introducing completely new materials, future developments might concentrate more on improving coating and production methods [25,26].

The production of femoral stems, acetabular cups, and articulating surfaces for hip implants is the main use of these cutting-edge materials. Because of their lower elastic modulus, Ti alloys such as Ti-15Zr-4Nb-4Ta and Ti-35Nb-7Zr-5Ta are very good at reducing stress on the surrounding bone. Longer implant lifespans and a lower risk of metal ion release are achieved by applying TiN and DLC coatings, bioactive layers, and metal–ceramic composites, which further improve these components’ wear resistance [10].

**Table 1:** Summary of the mechanical properties and clinical metrics of metallic materials employed for THA.

Material	Elastic Modulus [GPa]	Mechanical Strength [MPa]	Corrosion Resistance	Wear Resistance	Biocompatibility	Metal Ion Release	Cost
Stainless Steel	170–220	310–900	Low	High	Medium	Yes	Low
Co-Cr Alloys	190–280	620–1300	High	High	Medium	Yes	Medium
Ti Alloys	55–130	250–1200	High	Low	High	No	High
Oxidized Zirconium	90–100	400–500	High	High	High	No	Very High
Ta Alloys	2.5–3.9	50–110	High	Medium	High	No	Very High
Niobium Alloys	44–110	500–600	High	Low	High	No	Very High
SMA	30–90	800–1000	High	High	Medium	Yes	Very High

## 4. Polymers

The effectiveness and durability of implants have been significantly increased by the use of polymeric materials in THA. Significant progress has been made in limiting wear and related hazards, such as the creation of debris that can result in issues such as osteolysis, since the 1960s with the advent of polymer solutions [54]. These materials have been continuously improved throughout time to meet the rising need for implants that are more dependable and long-lasting. More sophisticated materials with improved mechanical qualities and human-body compatibility have been developed in recent decades as a result of research, with significant advancements occurring in the 2000s [55]. These developments have helped decrease long-term problems, increase the longevity of implants, and greatly enhance the quality of life of patients having THA.

**Figure 4:**
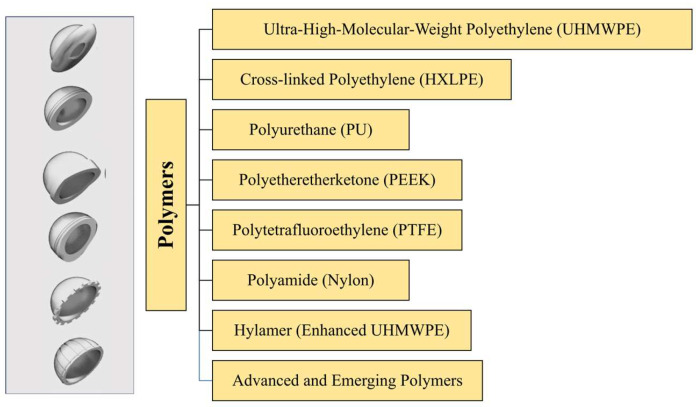
Main polymeric materials employed for THA.

The details of the polymeric materials used in THA (Figure 4) are provided in the next sections, followed by Table 2, which reports the main mechanical properties and clinical metrics. Moreover, Table S2 summarizes the main benefits, drawbacks, and applications of metallic materials.

### 4.1. Ultra-High-Molecular-Weight Polyethylene (UHMWPE)

In order to replace Polytetrafluoroethylene (PTFE), which had shown significant wear issues, Sir John Charnley created UHMWPE, a high-density, semi-crystalline polymer, in the 1960s. The structure of UHMWPE is composed of long chains of ethylene (−C_2_H_4_−)_n with a crystallinity of 50–55%, which adds to the material’s exceptional wear resistance and durability. These features make UHMWPE particularly suitable for THA. The use of UHMWPE greatly extended implant longevity by reducing friction and the frequency of revision surgery [9,15,54,55].

The primary use of UHMWPE in THA is in acetabular cups, which reduces the risk of revision surgery, improves implant longevity, and reduces friction between the femoral head and the acetabulum [10,13,17]. UHMWPE has disadvantages, chief among them being its susceptibility to oxidation, which can lead to the formation of wear debris, despite its outstanding biocompatibility and remarkable wear resistance. Implant loosening and ultimately failure may be caused by osteolysis triggered by this debris. In order to improve oxidative stability and reduce wear rates, current advancements include the introduction of other kinds of materials, e.g., HXLPE. Compared to traditional UHMWPE, HXLPE may be less resilient and more susceptible to fatigue and breaking [17,55].

The mechanical characteristics of UHMWPE, particularly its resistance to wear, are greatly influenced by its microstructure. Osteolysis is one of the consequences that can result from wear debris produced by the substance. The microstructure of the polymer is greatly influenced by its processing, which in turn impacts how well the material performs under load [54]. For example, sterilization procedures improve the material’s resilience to wear since they encourage the cross-linking of the polymer chains. The longevity of the implant may be jeopardized, however, as this process may also cause oxidative deterioration. One important tactic to combat free radicals and prolong the lifespan of UHMWPE-based implants has been the use of vitamin E as an antioxidant [11,13,19].

UHMWPE continues to be one of the most popular materials for THRs worldwide because of its clinical performance, processing ease, and mechanical strength balance. Although it has less wear resistance than these more recent materials, UHMWPE provides a reasonable cost–performance balance when compared to alternative materials like HXLPE and ceramics [23,55,56]. Recent studies have concentrated on using nanocomposites, such as those containing Carbon Nanotubes (CNTs), to further enhance the material’s mechanical qualities and resistance to wear. Furthermore, surface modification methods like nylon coatings and ion implantation are being investigated to improve UHMWPE’s biocompatibility and lessen wear, therefore increasing implant longevity [55].

### 4.2. Highly Cross-Linked Polyethylene (HXLPE)

In orthopedic surgery, HXLPE is frequently used, especially when making joint replacement components. Cross-linking polyethylene, which is mostly accomplished by subjecting the material to ionizing radiation like gamma rays, greatly improves the material’s resistance to wear and lowers the generation of wear particles, which are frequent problems in traditional polyethylene prostheses [10,57]. HXLPE, which is produced through radiation-induced cross-linking, constitutes the majority of highly cross-linked polyethylene used in THA. Wear resistance and oxidation stability are further improved by subsequent thermal treatments, such as annealing and remelting, which reduce the concentration of free radicals. As discussed for UHMWPE, a valuable strategy to increase implant durability is to enhance HXLPE’s oxidative stability. This goal can be achieved by adding antioxidants, such as vitamin E [10,21].

The introduction of HXLPE in the 1990s marked a significant advancement over traditional UHMWPE, offering greater wear resistance and reduced wear debris production, thus extending implant longevity. This development built upon earlier innovations, such as Sir John Charnley’s introduction of low-friction UHMWPE in 1958, which laid the foundation for modern hip arthroplasty. The transition from UHMWPE to HXLPE was driven by the need to address earlier materials’ wear and longevity issues [10,57]. HXLPE is primarily used in the production of acetabular liners for hip prostheses. This material is chosen for its high wear resistance, which is essential for articulating surfaces that must withstand heavy loads and repetitive movements. Acetabular cups made of HXLPE, combined with femoral heads of various diameters, contribute to improved implant stability and a reduced risk of dislocation. Second-generation HXLPEs, including sequentially irradiated and annealed varieties, have demonstrated encouraging results in short- to mid-term follow-ups, especially when combined with antioxidants [10].

The positive characteristics of HXLPE include significantly higher wear resistance compared to uncrosslinked polyethylene (PE), improved oxidative stability due to thermal treatments, and a significant reduction in the production of wear particles. The introduction of technologies such as chemically bonded Poly(2-methacryloyloxyethyl phosphorylcholine) (PMPC) grafting has further enhanced the material’s anti-wear properties [21]. However, there is a potential downside in that the high density, obtained through cross-linking, may compromise mechanical properties, such as hardness and rigidity. Oxidation during storage can degrade the material, reducing implant longevity. While increased radiation for sterilization in HXLPE raises some concerns, long-term studies suggest that these do not significantly impact clinical outcomes. Challenges remain, particularly concerning the risk of fatigue fractures and oxidation in certain HXLPE designs, especially with thin liners. Recent advances in antioxidant-infused HXLPE have further reduced oxidative degradation, extending implant longevity and improving clinical outcomes. The balance between enhancing oxidative stability and maintaining mechanical properties remains a key area of focus.

HXLPE is used globally in hip prostheses, especially in regions where implant longevity and the minimization of wear-related complications are of primary importance. The combination of HXLPE with larger diameter femoral heads is particularly widespread, improving implant stability and reducing the risk of postoperative complications such as dislocation [58]. Compared to conventional materials like Pe, HXLPE offers greater wear resistance and a lower production of wear particles, thereby reducing the incidence of osteolysis and aseptic loosening. However, compared to ceramic materials like alumina (Al_2_O_3_) and zirconia (ZrO_2_), HXLPE may offer greater cyclic load tolerance but might have a shorter useful life if not properly stabilized. The ongoing challenge lies in balancing mechanical properties with wear resistance, a focus of future developments in polymeric materials for joint arthroplasty.

### 4.3. Polyurethane (PU)

In orthopedic applications, PU is acknowledged as a versatile and promising biomaterial, especially in THA. Because of its exceptional mechanical qualities, such as its elasticity, flexibility, and biocompatibility, PU is a good choice for joint replacements when wear resistance and dynamic loading are crucial considerations [59,60].

Stress concentration on the implant and surrounding bone tissue is less likely thanks to PU’s elastic nature, which enables it to absorb and distribute stresses more uniformly. This is especially helpful with THA because mobility causes a lot of stress on the hip joint. PU is a desirable alternative for joint surfaces because of its capacity to replicate the mechanical characteristics of natural cartilage, resulting in smoother articulation and possibly less wear [60,61].

PU’s ability to be customized is one of its main benefits. Depending on the particular needs of the implant design, the material can be developed to display a variety of mechanical qualities, from soft and flexible to more rigid. This adaptability makes it possible to design implants that are specific to each patient’s requirements, which could enhance THA results [59,62].

But even though PU has a lot to offer, there are some drawbacks. The material’s long-term endurance is a major problem, especially given the cyclic stress conditions common in hip joints. PU may deteriorate with time, resulting in a decrease in mechanical strength and perhaps implant failure. Furthermore, although PU is usually biocompatible, studies are still being conducted to determine how it will interact with biological tissues over the long term, including the possibility of inflammation or negative reactions [62].

PU is still being investigated as a material for acetabular liners and other THA components despite these difficulties. It is a very versatile material for a variety of orthopedic applications due to its capacity to be molded into numerous shapes, such as solid articulating surfaces or porous scaffolds [62].

PU provides a special blend of elasticity and biocompatibility that could improve implant function, especially in terms of lowering wear and enhancing patient comfort, when compared to more conventional materials like metal alloys or ceramics. Although metals provide strength and endurance and ceramics offer improved hardness and wear resistance, PU’s flexibility enables better shock absorption and stress distribution, which may result in implants that last longer [59,62].

### 4.4. Polyetheretherketone (PEEK)

A high-performance polymer called PEEK has drawn a lot of interest in the field of orthopedic implants, especially in THA. PEEK is becoming more and more popular as a substitute for conventional materials like metals and ceramics in joint replacements because of its exceptional mechanical qualities, which include high tensile strength, biocompatibility, and chemical resistance [17,18].

In THA applications, PEEK’s mechanical qualities are especially beneficial. Compared to metals like Ti, its modulus of elasticity is more similar to that of human bone, which helps to lessen stress shielding, a typical problem with metallic implants that can cause bone resorption. Because of the material’s radiolucency, post-operative imaging is made easier, which is essential for tracking the health of the implant and the surrounding bone tissue [63].

The ability of PEEK can be reinforced with fibers, such as carbon fibers, to increase its mechanical strength, and wear resistance is one of its many noteworthy benefits. With its enhanced wear characteristics, Carbon-Fiber-Reinforced Polyetheretherketone (CFR-PEEK) is a composite material that is being investigated for usage in load-bearing orthopedic applications, such as THA. Revision operations may be less necessary with CFR-PEEK since it may give implants a longer lifespan [64,65].

Nevertheless, PEEK has drawbacks in addition to its benefits. Although the material’s wear resistance is superior to that of some polymers, it is still inferior to that of metal alloys or ceramics, which restricts its use in several high-load applications. Furthermore, because of its semi-crystalline structure that is sensitive to temperature, PEEK exhibits crucial processing operations [66]. The overall cost of implants made of this material may rise as a result of its comparatively high production costs.

PEEK is being investigated mainly for acetabular liners and femoral stems in the context of THA. It is a good fit for these components due to its mechanical qualities and biocompatibility, but research is still being carried out to increase its resistance to wear and long-term performance under cyclic loading circumstances [22,56].

PEEK has a special blend of mechanical, biocompatible, and radiolucent qualities that can improve patient outcomes when compared to more conventional materials like metals and ceramics. PEEK is a promising material for future orthopedic applications because of its bone-like modulus and the possibility of customization through reinforcement, even if metals offer greater strength and ceramics offer outstanding wear resistance [10,13].

### 4.5. Polytetrafluoroethylene (PTFE)

Teflon, also referred to as PTFE, was one of the earliest polymers utilized in orthopedic implants, especially in THA. Because of its low coefficient of friction, superior chemical resistance, and biocompatibility, PTFE was first preferred for use in hip prosthesis articulating surfaces [56,67].

Over time, however, clinical results showed notable limitations. Under the high-stress, repeated circumstances of the hip joint, PTFE showed poor resistance to wear, which resulted in rapid deterioration and the formation of wear particles [12,67]. Inflammatory responses and osteolysis—bone resorption that results in implant loosening and failure—were frequently brought on by these particles [10,17]. Revision operations were often necessary for such issues.

Because of these problems, PTFE was gradually replaced by more sophisticated polymers that provide superior wear resistance and have demonstrated better long-term results, like PEEK and UHMWPE. The lifespan of hip implants has been increased, and the incidence of osteolysis has been considerably decreased by these novel materials [17,56].

Although PTFE is no longer utilized in hip replacements today, it played a crucial part in the creation of the first orthopedic implants. It offered crucial information that guided the development of stronger and safer materials, paving the way for the biomaterials found in modern implants.

### 4.6. Polyamide (Nylon)

One of the first synthetic polymers investigated for use in THA was polyamide, also referred to as nylon. Strong mechanical qualities, such as high tensile strength, superior elasticity, and wear resistance, attracted attention to nylon [68]. Because of these qualities, it was a desirable option for the articulating surfaces of hip implants, where minimizing wear and friction is essential to implant longevity.

At first, acetabular liners and other bearing surfaces for hip prostheses were made of nylon. Its toughness and flexibility were thought to be beneficial in the development of long-lasting, wear-resistant joint components that could tolerate the hip joint’s cyclic loads. Nylon implants performed adequately in the short term, according to promising early clinical outcomes [68].

But as time went on, several serious problems emerged. Because nylon collects moisture from its surroundings, including physiological fluids, it is hygroscopic [69]. Dimensional changes brought on by this absorption, including swelling and material softening, may jeopardize the implant’s fit and functionality. Furthermore, although initially sufficient, nylon’s wear resistance was insufficient under the high-stress circumstances of the hip joint. Like other early polymers used in THA, the material’s breakdown produced wear particles that triggered inflammatory reactions and osteolysis [70].

Because of these drawbacks, nylon was subsequently phased out of THA in favor of more sophisticated materials like PEEK and HXLPE, which provide better stability and wear resistance under load.

### 4.7. Hylamer (Enhanced UHMWPE)

The early 1990s saw the introduction of hylamer, an enhanced UHMWPE, as a cutting-edge material for THA. In order to increase wear resistance and prolong the life of hip implants, hylamer was specially designed to improve the mechanical qualities of regular polyethylene [18].

A novel technique of airborne gamma irradiation was used to process hylamer, which was thought to enhance the material’s characteristics by raising its molecular weight and cross-linking density. The goal of this procedure was to produce a material that would be more resilient to wear and tear than the typical polyethylene used in hip prostheses. Early clinical findings were encouraging, demonstrating shorter-term implant longevity and lower wear rates [18,64].

Long-term research and clinical follow-ups, however, showed serious disadvantages. Despite being designed to fortify Hylamer, the gamma irradiation method unintentionally caused the material to oxidatively degrade. Similarly to the problems seen with previous polyethylene materials, this deterioration led to a significant rise in wear rates over time, producing wear particles that induced inflammation and osteolysis. Higher rates of implant failure and the requirement for revision operations were caused by these issues [71].

The use of hylamer in THA was ultimately stopped as a result of these problems. It became evident that the material’s initial advantages were offset by its vulnerability to oxidative breakdown. Since then, hylamer has been replaced by more recent substitutes that provide better wear resistance and less oxidative degradation, like HXLPE [19].

### 4.8. Advanced and Emerging Polymers

With the creation of cutting-edge and novel polymers that seek to overcome the drawbacks of conventional materials, the area of THA is still developing. These cutting-edge polymers are intended to increase hip implant lifetime and function by decreasing oxidative deterioration and improving wear resistance.

The development of second-generation HXLPE is among the most noteworthy developments. The addition of additional processes, such as successive irradiation and annealing, and the introduction of antioxidants like vitamin E, as discussed in previous sections, have been applied to this material. These changes are designed to minimize wear rates and prevent oxidative deterioration, which were important concerns in prior versions of HXLPE [18,19].

Along with better HXLPE, resurfacing concepts that use cementless HXLPE components have advanced. By reducing concerns such as liner dissociation and HXLPE fracture, these designs seek to offer wear resistance comparable to that of conventional THA. The objective is to develop implants that more closely resemble the hip joint’s natural biomechanics in order to lower problems and enhance patient outcomes [58].

There have been notable developments in the idea of dual mobility systems as well. HXLPE inserts used in these systems offer exceptional wear resistance along with sufficient flexibility to tolerate impact pressures during femoral head insertion. Future polyethylene generations are thought to benefit from this dual mobility design, which could lower dislocation rates and increase implant stability [72].

The usage of monoblock cementless components composed of advanced HXLPE is also being investigated in preliminary investigations. By addressing some of the drawbacks of existing implant designs, these designs seek to reduce bone loss and enhance the implant’s long-term stability [73].

**Table 2:** Summary of the mechanical properties and clinical metrics of polymeric materials employed for THA.

Material	Elastic Modulus [GPa]	Mechanical Strength [MPa]	Oxidation	Wear Resistance	Biocompatibility	Debrits Release Risk	Cost
UHMWPE	1.0–1.5	25–40	High	High	High	Medium	Medium
HXLPE	0.6–1.2	35–50	Medium	High	High	Low	High
PU	0.2–0.7	10–60	Low	High	Low	Medium	Low
PEEK	4.0–7.0	80–100	Low	Low	Medium-high	High	High
PTFE	0.4–0.6	20–35	Low	Low	Medium-high	High	Medium
Nylon	1.0–3.0	60–80	Medium	Medium	Low	High	Low
Hylamer	2.5–3.0	80–100	High	Medium	Medium	Low	High

## 5. Ceramics

Because of their remarkable wear resistance and biocompatibility, ceramic materials have grown in significance in THA. Ceramics like ZrO_2_ and Al_2_O_3_ have been utilized for hip implant-bearing surfaces since the 1970s because they provide long-lasting, low-friction interfaces that reduce wear over time [23,74]. These materials are prized for their smooth surfaces and hardness, which lower wear particle generation, which is essential for implants’ long-term effectiveness [75].

More durable parts that can better endure the mechanical demands of the hip joint and offer superior fracture resistance have been made possible by advancements in ceramic technology [24,76]. Additionally, ceramics are used because of their physiologically inert surfaces, which lower the possibility of negative responses and guarantee compatibility with tissue and bone [77].

In order to prolong the life of hip implants, research is still being carried out to improve the mechanical qualities of ceramics, such as increasing fracture toughness and improving manufacturing processes. By reducing the need for revision operations and enhancing overall results, these advancements seek to give patients a dependable and long-lasting alternative [23,77].

**Figure 5:**
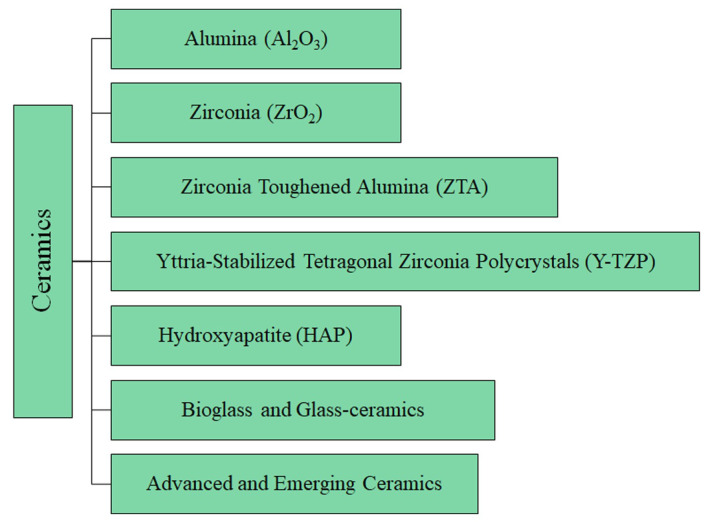
Main ceramic materials employed for THA.

The details of the ceramic materials (summarized in Figure 5) used in THA are provided below, followed by Table 3, which reports the main mechanical properties and clinical metrics. Moreover, Table S3 summarizes the main benefits, drawbacks, and applications of metallic materials.

### 5.1. Alumina (Al_2_O_3_)

Because of its low chemical reactivity, high hardness, wear resistance, and biocompatibility, Al_2_O_3_ is a ceramic material that is frequently used in THA. Because of these properties, Al_2_O_3_ is especially well suited for prosthetic articular surfaces, where it helps lower the risk of osteolysis and wear brought on by debris from prosthetic movement [9,11]. Al_2_O_3_ greatly lowers the risks of inflammation and debris formation, making it particularly useful in load-bearing applications like hip prostheses [27,76].

High-purity aluminum oxide, commonly referred to as bioinert alumina, makes up the majority of the substance. Al_2_O_3_’s mechanical qualities might be jeopardized by even minute impurities; therefore, cleanliness is crucial. Advanced processing methods such as Hot Isostatic Pressing (HIP), which raises Al_2_O_3_’s density and mechanical strength and increases its resistance to fractures, are frequently used to improve its performance [17,74]. By compacting the material under high pressure and temperature, HIP gets rid of any cavities or flaws that can cause problems.

Al_2_O_3_ was first used in orthopedics in the 1970s as a better substitute for metallic and polymeric materials, which were prone to severe wear and debris production, resulting in inflammation and osteolysis [11]. The durability and lifetime of hip implants significantly improved with the introduction of Al_2_O_3_. Third-generation ceramics, especially enhanced Al_2_O_3_ forms, were widely used by the 1990s due to advancements in ceramic technology [76]. Currently, Al_2_O_3_ is mostly utilized in the production of acetabular inserts and femoral heads, two essential parts of hip prosthesis. These parts are frequently utilized in ceramic–ceramic couplings or combined with other bearing surfaces, such as HXLPE. Excellent wear resistance is provided by these combinations, which is essential for the implant’s long-term success, especially in younger and more active patients [18,76,77]. Al_2_O_3_ can shorten implant life by lowering the risk of inflammation and osteolysis and ensuring little debris production when combined with polyethylene or other ceramics.

Furthermore, Al_2_O_3_’s biocompatibility is a significant benefit since the body tolerates it well, and its inertness guards against negative reactions that can result in implant failure. The use of Al_2_O_3_ in conjunction with Ti has become more popular, particularly for parts that need to be extremely strong and integrate well with bone. In load-bearing applications, where both materials function at their best, Ti works well with Al_2_O_3_ [76].

Al_2_O_3_ is used extensively around the world, but it is especially popular in Europe and Asia, where it is frequently chosen for younger, more active patients who need long-lasting, robust prostheses. The material’s ability to lessen wear and prolong implant life is what is driving this trend, which is significant in healthcare systems that place a high value on long-term results [13,21]. Al_2_O_3_ use is growing steadily in the US thanks to technical developments that have improved the accessibility and dependability of ceramic implants [76].

Al_2_O_3_ has several clear advantages over metallic materials like Co-Cr, such as much lower wear rates and less debris creation, which reduces the danger of osteolysis, which is a prominent cause of implant failure in metal-based systems [10]. However, because Al_2_O_3_ is more likely to fracture than metal components, its inherent brittleness necessitates careful handling during implantation. To guarantee ideal placement and lower the risk of problems, this calls for exacting surgical methods and careful preoperative preparation [17,78].

### 5.2. Zirconia (ZrO_2_)

ZrO_2_ is a ceramic material that is a good choice for THA because of its great strength, toughness, and biocompatibility. In applications where resistance to crack propagation is essential, ZrO_2_ outperforms other ceramics like Al_2_O_3_ due to its exceptional mechanical qualities, which include high fracture toughness [10]. Its special phase-transformation ability, called transformation toughening, is what gives the material its exceptional toughness. Under stress, the material changes phases, effectively halting the spread of cracks and increasing its longevity in load-bearing applications [21,22].

Small additions of Yttria (Y_2_O_3_) are commonly used to stabilize ZrO_2_ in its tetragonal phase at room temperature, producing Yttria-Stabilized Tetragonal Zirconia Polycrystals (Y-TZPs). Because pure ZrO_2_ changes phases at different temperatures, which could jeopardize the material’s structural integrity, this stability is crucial. Because of its exceptional strength and toughness, Y-TZP is especially well suited for use in acetabular and femoral heads in hip prostheses [9].

Due to ZrO_2_’s promising mechanical qualities and biocompatibility, it was first used in orthopedic implants in the 1980s [25]. ZrO_2_ outperformed other ceramics in terms of toughness, according to early research, and was able to tolerate the mechanical stresses of THA. Due to its great wear resistance and minimal friction when articulated against polyethylene or other ceramic materials, ZrO_2_ had gained popularity in orthopedic applications by the 1990s, especially in the production of femoral heads [19].

ZrO_2_ is frequently combined with other elements, including Al_2_O_3_, in contemporary THA to produce composite ceramics that capitalize on the advantages of both materials. These composites seek to create prosthetic parts that are long-lasting and durable by fusing the superior wear resistance of Al_2_O_3_ with the high toughness of ZrO_2_ [9,56]. ZrO_2_-based femoral heads have demonstrated encouraging outcomes in lowering wear and extending the life of hip implants when combined with HXLPE acetabular inserts [17,23].

Another important element in ZrO_2_’s effectiveness as a THA material is its biocompatibility. The material is a safe choice for long-term implantation because it is well tolerated by the body and does not cause negative tissue reactions due to its inert nature [9,18]. Additionally, the material’s low friction qualities—which are crucial for avoiding wear debris and the risk of osteolysis, a typical issue in Metal-on-Metal (MoM) and Metal-on-Polyethylene (MoP) articulations—are facilitated by its high density and smooth surface finish [17].

Nevertheless, ZrO_2_ has certain disadvantages in addition to its benefits. The possibility of phase change in vivo, which can eventually result in a reduction in toughness and an increase in wear, is one of the main issues [19]. In order to reduce this risk, ZrO_2_ and Al_2_O_3_ composites and other improvements have been developed. Furthermore, ZrO_2_ is extremely wear-resistant, but because of its brittleness—which is less noticeable than in pure Al_2_O_3_—it must be handled carefully during implantation to prevent fractures [21].

### 5.3. Zirconia-Toughened Alumina (ZTA)

An improved option for THA is ZTA, a composite ceramic material that combines the advantageous qualities of both ZrO_2_ and Al_2_O_3_. By adding ZrO_2_ particles, which work to stop cracks from spreading, ZTA is designed to increase the toughness of Al_2_O_3_, which is a major drawback of pure Al_2_O_3_ ceramics. This combination produces a material that benefits from ZrO_2_’s transformation toughening qualities while retaining the excellent hardness and wear resistance of Al_2_O_3_ [22,67].

Typically, ZrO_2_ particles are distributed across an Al_2_O_3_ matrix to form ZTA. The required toughening effect is provided by the ZrO_2_ concentration, which typically ranges between 15 and 25%. This makes ZTA more capable of absorbing and dissipating stress than pure Al_2_O_3_. The ceramic material produced by this composition has much better fracture toughness, which makes it ideal for high-stress applications like the femoral heads of hip prostheses [17,56].

In an effort to address the brittleness of conventional ceramic materials used in orthopedics, researchers started developing ZTA in the late 1980s and early 1990s. An important development was the addition of ZrO_2_ to Al_2_O_3_, which greatly increased its toughness while preserving the favorable qualities of Al_2_O_3_, such as its biocompatibility and resistance to wear [67]. ZTA was well known as the preferred material for high-performance orthopedic implants by the mid-1990s, particularly when fracture toughness and wear resistance were crucial.

ZTA is widely utilized in the manufacture of acetabular inserts and femoral heads in contemporary THA. Because of its special set of qualities, the material is perfect for younger, more active patients who put more strain on their prosthetic joints. HXLPE acetabular inserts are frequently used in conjunction with ZTA components, which further minimize attrition and increase implant longevity. Over time, the implant’s dependability is increased by the material’s capacity to experience stress-induced phase change, a property inherited from ZrO_2_, which offers an extra layer of protection against fracture propagation [11,21].

Another important component of ZTA’s performance as a THA material is its exceptional biocompatibility. The ZrO_2_ phase adds to the material’s overall toughness without sacrificing its biocompatibility, while the Al_2_O_3_ matrix guarantees a solid and inert surface that the body can tolerate. For long-term implantation in hip prostheses, ZTA is the perfect material due to its toughness and biocompatibility [18]. The use of ZTA in THA is becoming more and more common worldwide, particularly in Europe and Asia, where creating cutting-edge materials for younger patients is highly valued. ZTA is becoming more and more popular in the US when better mechanical qualities and durability are needed, especially for active patients who want the best performance from their implants [21].

ZTA does, however, have certain drawbacks in addition to its many benefits. ZTA’s widespread use may be constrained by its costlier and intricate manufacturing method compared to that of pure Al_2_O_3_ or ZrO_2_. Furthermore, ZTA still needs to be handled carefully during implantation to prevent fractures, even if it is less brittle than pure Al_2_O_3_ [67].

### 5.4. Yttria-Stabilized Tetragonal Zirconia Polycrystals (Y-TZPs)

Because of its remarkable mechanical qualities, Y-TZP is a kind of cutting-edge ceramic material that has gained significant importance in THA. The main constituent of Y-TZP ceramics is ZrO_2_, which is stabilized at room temperature in the tetragonal phase by the addition of Y_2_O_3_. Because it stops ZrO_2_ from spontaneously changing phases, which might result in structural defects, this stability is essential [22].

The exceptional strength and fracture toughness of Y-TZP ceramics are noticeably greater than those of pure ZrO_2_ or Al_2_O_3_. The ability of Y-TZP to experience transformation toughening, or stress-induced phase transformation, is the secret to its resilience. A localized increase in volume can occur when the tetragonal phase of ZrO_2_ in Y-TZP changes into the monoclinic phase under mechanical stress. In high-stress applications, such as the femoral heads in hip prostheses, this volume shift produces compressive stresses that seal cracks and stop them from spreading, increasing the material’s longevity [17].

The requirement for materials that could endure the mechanical demands of joint replacements sparked the creation of Y-TZP ceramics in the 1980s. Early research showed that Y-TZP was an excellent choice for orthopedic implants because it was more durable than other ceramics. Because of its exceptional wear resistance and minimal friction when articulated against polyethylene or other ceramics, Y-TZP became widely used in THA by the 1990s, especially for the fabrication of femoral heads [9].

Y-TZP ceramics are preferred in contemporary THA due to their exceptional strength, durability, and biocompatibility. These materials are frequently used with HXLPE inserts to produce low-wear articulations that increase the implant’s lifespan in femoral heads and acetabular components [17,22].

Another well-known quality of Y-TZP ceramics is their superior biocompatibility. The material is a safe choice for long-term implantation because it is extremely inert and does not cause negative tissue reactions. Furthermore, Y-TZP components’ smooth surface finish minimizes wear and friction, which is essential for reducing the production of wear debris and the danger of osteolysis, a typical consequence in MoM articulations [9].

Y-TZP ceramics do have certain drawbacks though. The possibility of Low-Temperature Deterioration (LTD), also referred to as aging, in which the material changes phase over time when exposed to bodily fluids, is a major worry [22,56]. The risk of implant failure may rise as a result of this deterioration since it may result in a reduction in mechanical qualities, including strength and toughness.

### 5.5. Hydroxyapatite (HAP)

Because of its exceptional osteoconductive and biocompatible qualities, HAP, also known chemically as Ca_10_(PO_4_)_6_(OH)_2_, is a bioceramic substance that is frequently utilized in orthopedic surgery, especially THA. Because HA closely resembles the mineral found in human bones, it works especially well to encourage bone formation and implant integration. Because of this resemblance, HA can help the implant and surrounding bone tissue form a solid bond, increasing the prosthesis’s durability and lifetime [13,18].

Researchers first investigated HA’s potential as a covering material for metallic implants in the late 1970s and early 1980s, which is when it was first used in orthopedics. The chosen option for improving osseointegration was HA because of its bioactive properties and capacity to form a link with bone. Usually, the substance is used as a coating on metallic implants, especially Ti and its alloys, by applying a thin, consistent layer of HA to the implant surface using plasma-spraying techniques [13,18]. By serving as a scaffold to promote the formation of new bone tissue, this covering guarantees a solid connection between the implant and the bone.

Another important characteristic of HA that improves its osteoconductivity is its porous nature. The implant is further stabilized by the growth of bone tissue into the HA coating, made possible by the porous design [18,19]. When it comes to hip replacements, where the implant needs to sustain heavy mechanical loads while still being securely attached to the bone, this feature is especially helpful.

HA is being investigated for usage as a covering, as well as in the creation of scaffolds and composite materials for bone regeneration. In order to generate materials that not only encourage bone growth but also offer mechanical support to weak or injured bone structures, these composites blend HA with polymers or other ceramics [13].

HA does have several drawbacks though. Because of its poor tensile and compressive strengths and intrinsic brittleness, the material is not a good choice for load-bearing applications when used alone. In order to get around this restriction, HA is frequently applied as a coating for implants as opposed to being a bulk material, or it is mixed with other materials to improve its mechanical qualities [56,61].

### 5.6. Bioglass and Glass-Ceramics

One kind of bioactive glass called “Bioglass” forms a direct bond with bone to encourage osteointegration and bone growth. The field of bioactive ceramics was founded in the late 1960s when Larry Hench created the first synthetic material to chemically connect with bone, the original 45S5 Bioglass^®^. Silica (SiO_2_), sodium oxide (Na_2_O), calcium oxide (CaO), and phosphorus pentoxide (P_2_O_5_) are commonly found in Bioglass. When Bioglass is exposed to bodily fluids, a hydroxycarbonate layer forms on its surface that resembles the mineral phase of bone, which makes it easier for osteoblasts to attach and for new bone to be deposited [79,80].

Glass-ceramics are polycrystalline materials made from glass that has undergone controlled crystallization. Because of their superior mechanical and thermal qualities, they can be used in a variety of biomedical applications, such as bone regeneration and repair. Because of the surface changes that take place after implantation, these materials can adhere to bone, resulting in the development of a stable apatite layer at the bone–material interface [79,81].

Bioglass’s main uses in THA are as a covering for implants to improve osteointegration and as a replacement for bone grafts to fill in deficiencies. Because Bioglass is bioactive, it can directly promote bone formation at the implant’s interface, increasing the prosthesis’s stability and durability. Conversely, glass ceramics serve a similar purpose but have more mechanical strength, which makes them appropriate for load-bearing applications where pure Bioglass could be too brittle [80,81].

However, glass ceramics and Bioglass have disadvantages despite these advantages. Because of its brittleness and low mechanical strength, Bioglass can only be employed in non-load-bearing applications or as coatings in place of bulk materials. Glass ceramics are strong, but they nevertheless require cautious handling during implantation to prevent fracture, especially under severe stress [75,80].

### 5.7. Advanced and Emerging Ceramics

New ceramic materials that promise to improve biomechanical performance, durability, and bone integration are being introduced as THA continues to advance. Biolox Delta, gene-activating glasses, nanoceramics, and porous Bioglass are some of the most cutting-edge and inventive materials being developed or utilized today. These materials have unique benefits that have the potential to completely transform hip prostheses in the future.

In contrast to conventional ceramics like pure Al_2_O_3_, Biolox Delta is a cutting-edge ceramic designed to increase hardness and fracture resistance. By combining Al_2_O_3_ and Y-TZP, this material increases mechanical strength and lowers the possibility of disastrous in vivo fractures. One of the most cutting-edge ceramics utilized in joint prostheses at the moment is Biolox Delta, which offers a notable improvement in implant endurance and safety [82]. Because of its increased strength, it is especially appropriate for young, energetic patients who need implants that can endure heavy, repeated loads.

One fascinating development in the field of bioactive ceramic materials is gene-activating glasses [83]. These materials, which are currently in the research and development stage, have the potential to provide a new degree of bioactivity by triggering particular cellular responses that could improve tissue regeneration in addition to encouraging bone growth [83]. These glasses could revolutionize the design and usage of orthopedic implants by facilitating quicker healing and better bone integration if their potential can be realized in clinical settings. With its goal of more intricate interactions with bone tissue, this innovation represents a more advanced frontier than conventional bioactive materials.

A new field of study called nanoceramics aims to enhance cell adhesion and proliferation while encouraging bone formation at the nanometric level. Among these materials are nano-alumina formulations, which have a great deal of promise for enhancing the biomechanical functionality of prosthetic implants. Compared to traditional ceramics, nanoceramics provide the potential to improve stress distribution and integration with bone while lowering the risk of fractures [56,61]. Nanoceramics, as opposed to Biolox Delta, concentrates on fine-tuning the interaction between the implant and the biological environment.

Through in vivo mineralization and bone ingrowth into the implant’s pores, porous Bioglass is being developed to enhance bone regeneration. These materials show promise for future uses where high bioactivity and mechanical strength are required, especially for implants that need to integrate with the bone effectively [23,79]. Porous Bioglass seeks to strike a balance between bioactivity and the capacity to support intricate bone structures and fluctuating mechanical loads, in contrast to other cutting-edge ceramics.

## 6. Composites

Composite materials are increasingly essential in THA due to their ability to combine the capabilities of diverse materials, resulting in implants that are both strong and lightweight. These materials are perfect for the needs of the human body because they provide a blend of high mechanical strength, flexibility, and biocompatibility [66,84,85].

The capacity of composites to be customized to meet certain mechanical and biological needs is a significant benefit. In order to preserve bone health surrounding the implant, fiber-reinforced composites can improve stress distribution and decrease stress shielding by imitating the mechanical behavior of natural bone [64,66]. Because of their versatility, composites are very appealing for THA, where striking a compromise between strength and flexibility is essential [86].

In order to improve performance and durability, recent research has focused on optimizing composite composition and production. In order to create a more dependable and long-lasting prosthesis, efforts are being made to improve the implant’s stress distribution and fiber–matrix bonding [66,85]. Hip implants that provide longer-lasting solutions with fewer issues are becoming possible thanks to these improvements, which will ultimately improve patient outcomes [64,85,86].

**Figure 6:**
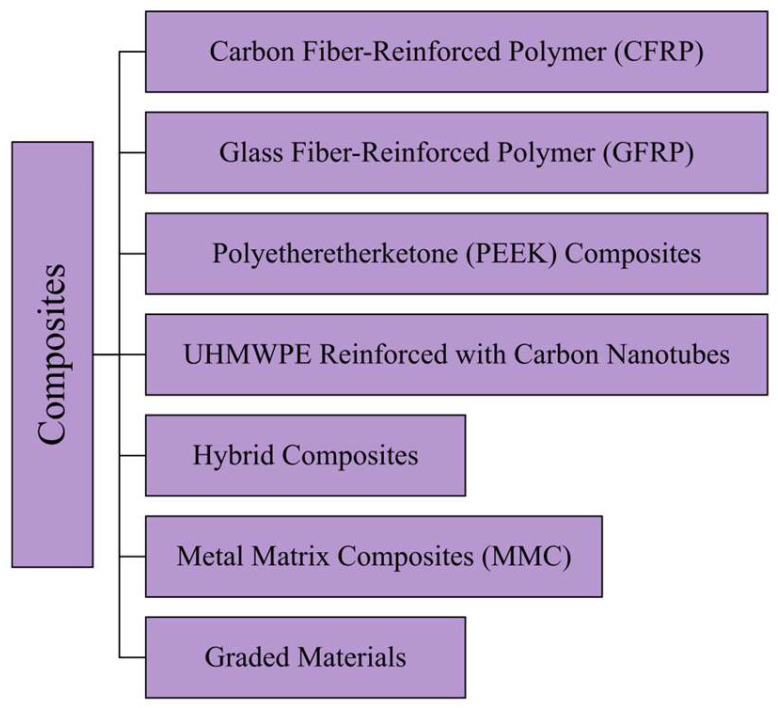
Main composites employed for THA.

The details of the composite materials (see Figure 6) developed for THA are provided below, followed by Table 4, which reports the main mechanical properties and clinical metrics. Moreover, Table S4 summarizes the main benefits, drawbacks, and applications of metallic materials.

### 6.1. Composite and Reinforced Materials

The biomechanical performance of implants used in THA has been improved by the development of composite materials, especially those reinforced with carbon fibers, such as CFR-PEEK. These materials are lightweight, highly biocompatible, and have a high mechanical strength. They are especially good at minimizing “stress shielding”, which can lead to bone resorption in the vicinity of the implant. The mechanical characteristics of the implant can more closely resemble those of natural bone because of this decrease in stress shielding, which helps mitigate problems caused by stiffness differences between the prosthesis and bone tissue [9,64].

A PEEK polymer matrix reinforced with carbon fibers makes up CFR-PEEK. In terms of mechanical performance and biocompatibility, this combination produces a material that is both robust and lightweight, providing a better option than conventional materials [9,64]. Because of these characteristics, CFR-PEEK is especially well suited for orthopedic applications where strength and flexibility must be balanced.

The first CFR-PEEK prostheses were developed in the 1980s and 1990s, marking the beginning of the use of composite materials in THA [9].

Overall, the use of composites has increased dramatically over the last few decades because of advancements in manufacturing technologies and a growing need for high-performance prosthetics that are appropriate for patients who are younger and more active [9,84]. Early composite prostheses showed notable advantages in lowering stress shielding and enhancing load distribution, such as those composed of polyetherimide (PEI) reinforced with carbon and glass fibers [66].

The majority of composite materials are utilized in the production of THA femoral stems, which gain the most from the decreased stiffness and enhanced mechanical compatibility that composites provide [9,64]. Composites are occasionally used in the acetabular cup as well, frequently in conjunction with other materials to maximize implant stability and wear resistance [84].

Other composite materials utilized in THA include Metal Matrix Composites (MMCs) and Fiber-Reinforced Ceramic Composites (FRCCs), in addition to CFR-PEEK. Fiber reinforcements like HAP are used to improve the mechanical properties of ceramic-polymer composites. This results in a robust, wear-resistant material that can be used in prosthetic applications [25]. MMCs, which are less prevalent in THA, entail adding fibers or other materials to metal matrices to increase their mechanical strength and resistance to wear. Because of their strong mechanical qualities, these composites have potential benefits in orthopedic applications, although they have mostly been studied in broader contexts [23].

### 6.2. Advanced, Innovative, and Futuristic Composite and Reinforced Materials

With the creation of novel composite and strengthened materials, the field of THA is undergoing tremendous progress. By overcoming the drawbacks of conventional materials like metals and ceramics, these materials are intended to improve the biomechanical performance, longevity, and biocompatibility of hip implants [9,64].

To push the limits of what is feasible in orthopedic implants, researchers are actively investigating novel fiber-reinforced ceramics, MMCs, and polymer composites. These materials are being actively investigated and improved for possible therapeutic uses, thus they are not merely theoretical [25,66].

The creation of polymer composites, such as UHMWPE reinforced with HAP and CNTs, is one of the most exciting new fields. In addition to having outstanding biocompatibility, these composites are being designed to provide improved mechanical qualities, such as increased tensile strength and wear resistance [84]. For example, the goal of adding CNTs is to strengthen the implants’ structural integrity and increase their resistance to the mechanical stresses they experience over time [9].

Carbon-Fiber-Reinforced Polymers (CFRPs) are another topic of interest because of their remarkable mechanical durability. The light weight and strong strength of CFRPs make them especially attractive because they can minimize the possibility of stress shielding, a typical problem with conventional implants, by greatly reducing the stress on surrounding bone tissue [64]. Research is still being conducted on these materials to optimize their performance for clinical usage, but they are still in the exploratory stage [9].

Additionally, hybrid composites are becoming more popular. These materials generate a composite with improved mechanical qualities by combining various fiber types, such as basalt and carbon. The objective is to create materials with improved load distribution and greater durability that not only match but surpass the performance of currently available choices [64,66].

In THA, Functionally Graded Materials (FGMs) are an especially novel strategy. FGMs are composite materials for which their structure or composition varies gradually over the material’s volume. This gradient, which closely resembles the natural gradation present in human bone, can be created to maximize the mechanical qualities at various locations within the implant [87,88]. An FGM-made femoral stem, for instance, might change from having a harder core to having a more flexible surface, which would improve load distribution and lessen stress concentrations [87]. Because of this feature, FGMs are particularly helpful in reducing stress shielding, a problem that frequently arises with conventional implants that have uniform material properties [88].

Another novel strategy is MMCs. In order to obtain better mechanical qualities, such as increased strength and resistance to wear, these composites combine metal matrices with reinforcing components [23]. Although MMCs have long been employed in sectors such as aerospace, their usage in THA is a relatively recent and fascinating development. With the potential to provide durable solutions that withstand wear and mechanical degradation, researchers are investigating how these materials might be tailored to the particular requirements of orthopedic implants.

The potential of FRCCs in THA is also being studied. These composites are made to closely resemble the characteristics of genuine bone, frequently using ceramic matrices supplemented with fibers like HAP. In order to lower the chance of rejection and increase the overall success rate of procedures, the goal is to develop implants that blend in perfectly with the body [25]. These materials are interesting options for upcoming implant designs because of their mechanical strength and biocompatibility.

**Table 4:** Summary of the mechanical properties and clinical metrics of composites materials employed for THA.

Material	Elastic Modulus [GPa]	Mechanical Strength [MPa]	Chemical Reactivity	Wear Resistance	Biocompatibility	Debrits Release Risk	Cost
CFRPs	70–150	600–1500	Low	High	Medium	Medium	High
GFRPs	20–50	200–900	Low	Medium	Medium	Medium	Medium
PEEK-composites	12–150	80–120	Very Low	High	High	Low	High
UHMWPE + CNTs	1–5	25–50	Low	High	High	Medium	Medium
HAP Coated Composites	3–20	50–100	High	Medium	Very High	Low	Medium
CMCs	150–400	100–400	Very Low	Very High	Medium	Low	High
MMCs	70–300	200–1000	Variable	High	Medium	Medium	High
Graded Materials	10–200	100–1000	Variable	Variable	Very High	Low	Very High

## 7. Materials Comparison

The materials employed in THA are widely used and vary significantly. A direct comparison between all materials is not always possible, as their applications may differ depending on the specific prosthetic component. For example, stainless steel can be successfully used for stem fabrication but is unsuitable for the acetabular cup, femoral head, or liner. On the other hand, more advanced solutions—such as graded materials—can be effectively applied to all prosthesis components.

With the aim of comparing all analyzed materials, the first step was to classify them according to their application. The results are presented in Table 5. Materials that are no longer in use for THA—such as nylon—have been excluded from the analysis.

**Figure 7:**
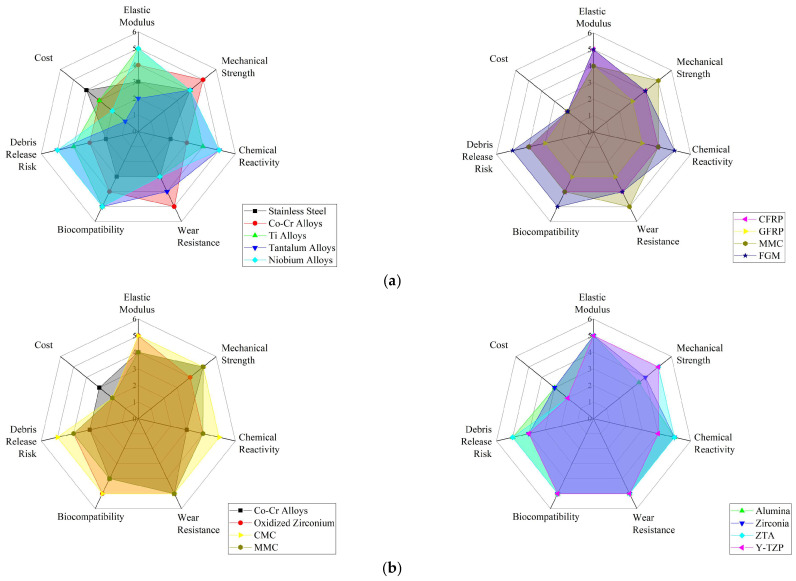

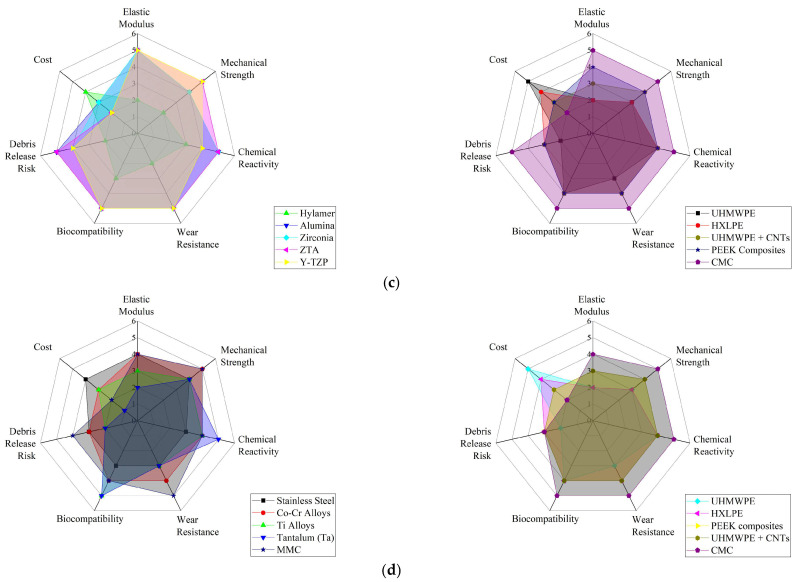
Comparison between materials employed for THA components: (**a**) stem, (**b**) femoral head, (**c**) liner, and (**d**) acetabular cup.

Starting from each component, it is then possible to compare different materials based on the main properties summarized in the previous tables. This allows for a comparative evaluation of each material, facilitating the selection of the most appropriate compromise for a specific application. The results are illustrated as a radar chart in Figure 7. The scores attributed to each material (ranging from 1 to 5) were based on a comparative analysis of the literature and existing clinical data. The evaluation considered quantitative properties such as elastic modulus and mechanical strength, as well as qualitative aspects like biocompatibility, chemical reactivity, and clinical reliability. Materials were scored relative to one another, with 5 representing excellent performance for a specific property and 1 indicating poor or suboptimal performance. As regards cost, a low score, e.g., 1, is related to an expensive material. When necessary, conservative estimates were made for emerging or composite materials based on experimental studies and reported trends.

The collected data allow us to draw the following conclusions:

- Metals offer good performance at relatively low costs, enabling broader accessibility. However, the main drawback of this material class is chemical reactivity, specifically the risk of metal ion release. When higher chemical stability is required, more expensive options, such as tantalum, must be considered.
- Polymers are less expensive and exhibit low wear coefficients, making them preferable for bearing surfaces. However, their limited mechanical properties restrict their use in structural components, such as the stem.
- Ceramics are highly valuable due to their superior wear resistance and chemical stability. However, they are more expensive and, because of their brittleness, are susceptible to debris formation and catastrophic failure.
- Composites display a wide range of characteristics depending on their constituents. Generally, the better their performance, the greater their complexity and, consequently, their cost.
- In general, improved material performance is associated with higher costs. Therefore, even if certain materials may be technically superior, they cannot be universally applied due to economic and practical constraints.

Overall, it is clear that no single material can be considered the optimal solution in every case. Material selection must take into account a variety of patient-specific factors, such as lifestyle, age, health conditions, and other variables, that cannot be generalized.

## 8. Cement Usage in THR

A fundamental method in orthopedic surgery, cementation in THA offers a strong and long-lasting way to attach implants to bone. Sir John Charnley’s pioneering work in the 1960s, which introduced the use of Polymethylmethacrylate (PMMA) bone cement, helped popularize this method. The foundation for contemporary cementation procedures was laid by Charnley’s 1958 and 1960 methods, which focused on using PMMA as a grout rather than an adhesive [89].

**Figure 8:**
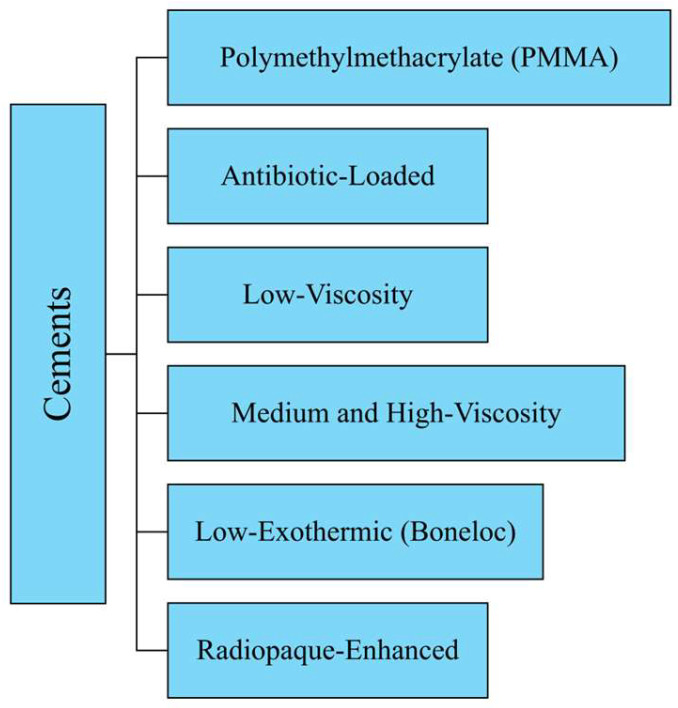
Main cements employed for THA.

**Figure 9:**
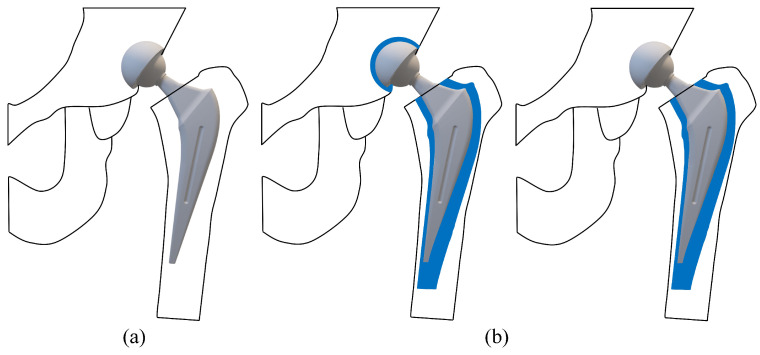
Cement application in THA: (**a**) cementless and (**b**) cemented.

Historically, PMMA has been the cornerstone material for cementation in THA. Over time, its composition has been enhanced with various additives, including antibiotics such as gentamicin, introduced in the 1970s to significantly reduce postoperative infections [89]. A summary of the main solution developed over the years is reported in Figure 8. Moreover, cement could be employed to fix different interfaces, as shown in Figure 9.

Techniques like vacuum mixing, more commonly used since the late 1980s, have been developed to reduce porosity in the cement, improving its mechanical properties and durability. These advancements allow better penetration into cancellous bone, ensuring a more secure fixation [90].

PMMA cement is created by mixing a liquid methylmethacrylate monomer with a powdered polymer that includes an initiator like benzoyl peroxide and a stabilizer such as hydroquinone, which prevents premature polymerization [90]. The polymerization process is exothermic, releasing heat that can cause thermal necrosis if not carefully managed [91]. Since the 1980s, modern cement has been tailored to specific surgical needs, with different viscosities available to improve bone penetration and mechanical interlock between the cement and bone [92]. Low-viscosity cements, developed in the 1980s, enhance penetration into cancellous bone, improving the implant’s overall stability, which is critical for long-term success. Additionally, antibiotic-loaded cements, like those incorporating gentamicin, provide an extra layer of protection against infections, especially in revision surgeries [11]. In recent years, the so-called biodegradable cements have been introduced. Poor biodegradability can lead to complications such as intramedullary hypertension. Therefore, the development of bone cements with improved biodegradability is crucial for biomedical applications.

Biodegradable orthopedic materials provide mechanical support and promote both osteoconductivity and osteoinduction at the implantation site, thereby contributing to bone tissue regeneration [93,94]. These materials gradually degrade within the body through mechanisms, such as dissolution, enzymatic degradation, and cellular phagocytosis, at a rate compatible with new bone formation.

As tissue fills the defect, the degradation by-products are absorbed or eliminated, and the implanted material is ultimately replaced entirely by newly formed bone, leaving no residue in the body.

Several types of cement have been used over time in THA:

- PMMA, the most commonly used bone cement, introduced by Charnley in the 1950s, became the standard by 1960. It is enhanced with additives like antibiotics and radiopaque agents for improved functionality [89,91,95];
- Antibiotic-loaded cement, a modified version of PMMA with added antibiotics like gentamicin, was introduced in the 1970s to reduce postoperative infections, commonly used in revision surgeries [89,91];
- Low-viscosity cement was developed in the 1980s for better penetration into cancellous bone, enhancing the interface between bone and cement [90,95];
- Medium and high-viscosity cement offers various handling options for surgeons, chosen based on specific surgical needs [95];
- Low-exothermic cement (Boneloc) was developed in the 1990s to reduce the heat generated during polymerization, though with less favorable mechanical properties compared to conventional PMMA [95];
- Radiopaque-enhanced cement, containing radiopaque agents like barium sulfate, was introduced in the 1970s to improve radiographic visualization, crucial for post-operative monitoring [89,91].

The primary advantage of using cement in THA is the immediate fixation it provides, allowing early mobilization and weight-bearing properties, particularly beneficial for elderly patients or those with poor bone quality [96]. However, potential drawbacks include long-term complications such as aseptic loosening, where the bond between the cement and bone weakens over time, leading to implant failure. Additionally, the exothermic reaction during cement curing poses a risk of thermal damage to surrounding tissues [91]. Despite these risks, cemented THA remains a preferred method in many clinical scenarios due to its reliability in achieving immediate stability and its suitability for patients with compromised bone quality, particularly in revision surgeries where bone integrity is often compromised [95].

Globally, the use of cemented versus uncemented implants varies significantly. In Europe, particularly in the UK, cemented THA is still prevalent, especially among older patients who benefit from the immediate stability that cemented implants provide [95]. In contrast, the United States has seen a trend towards uncemented implants since the early 2000s, favored for their potential to achieve biological fixation through bone ingrowth [96]. However, cemented THA remains crucial where immediate stability is paramount, continuing to show excellent long-term outcomes in various international studies.

Table 6 provides a clear and concise overview of the main characteristics of each type of cement used in THA. A summary of the main characteristics of cements is reported in Table S5.

## 9. Bearing Surfaces in THA

The bearing surfaces in THA are crucial for ensuring the longevity of the implant and the well-being of patients. The friction and wear resulting from the relative movements between these surfaces are one of the main causes of long-term prosthesis failure. If not managed correctly, these phenomena can lead to an increase in particle release, causing inflammation and osteolysis. This may, in turn, require revision surgeries, increasing the risk for the patient and the cost of treatment. In recent decades, research has led to the development of new materials and bearings that aim to reduce these negative effects and improve the overall performance of implants [47,60,97].

**Figure 10:**
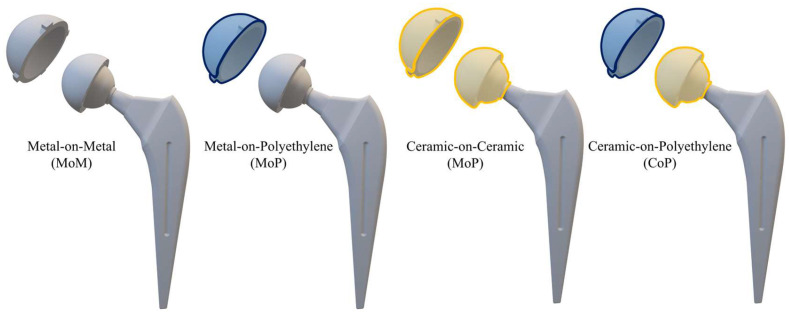
Widespread bearing surfaces for THA.

When analyzing the materials used for bearings in THA, the main focus is on the direct contact surfaces, namely the femoral head and the acetabular insert. These components are primarily responsible for the tribological behavior of the implant, largely determining the management of friction, wear, and particle release. The acetabular cup, while a crucial component of the prosthesis, primarily plays a structural and supportive role, providing implant stability and promoting bone integration, but with a lesser impact on overall tribology [82]. Consequently, research and innovation are primarily focused on the materials and technologies that can improve the performance of these contact surfaces by reducing wear and increasing biocompatibility. A graphic representation of the most commonly used bearing surfaces is reported in Figure 10, while the main benefits, drawbacks, and applications are summarized in Table 7.

**Figure 11:**
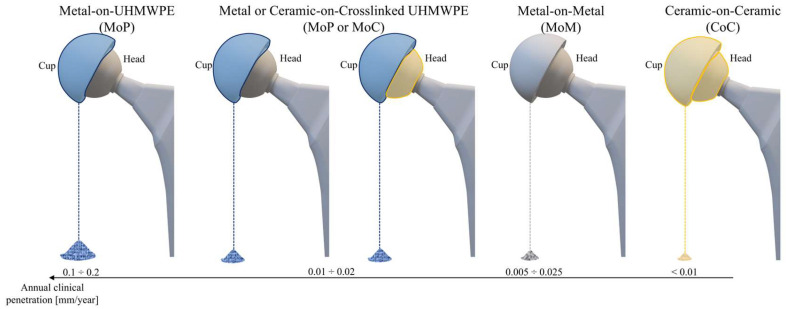
Annual critical penetration for main bearing surfaces [98].

Historically, Metal-on-Polyethylene (MoP) has been one of the first bearing combinations used in THA, thanks to its combination of strength and shock absorption capacity. Over time, polyethylene has been improved, evolving from conventional polyethylene to HXLPE, and designed to reduce wear (Figure 11). These developments have significantly reduced the oxidation and mechanical degradation problems associated with traditional polyethylene, making it still a very popular option, especially in older patients, where the implant’s workload is lower [47]. However, in younger and more active patients, where implant longevity is a priority, there is growing interest in using antioxidant-stabilized polyethylene, such as vitamin E, which promises to further improve material durability [32,47,97], following an approach similar to that discussed in the polymer sections.

The Ceramic-on-Polyethylene (CoP) combination was introduced to improve wear resistance compared to MoP, using a ceramic head combined with a polyethylene acetabular insert (see Figure 9). This combination has become particularly popular for its ability to reduce wear and debris production and for being a viable alternative for patients with metal allergies. Advanced ceramics, such as Biolox Delta, which combine ZrO_2_ particles stabilized with Y_2_O_3_, strontium, and chromium oxide, represent a further step forward in terms of mechanical strength and reduced fracture risk [56,60,82].

The Metal-on-Metal (MoM) combination, introduced with the intent to further reduce wear compared to MoP, has seen a drastic decline due to the issues associated with metal ion release and adverse reactions in soft tissues. Although this combination was popular in the past, the risk of serious complications, such as pseudotumors and damage to surrounding soft tissues, has led to a significant reduction in its use [32,49,98]. Currently, the use of hybrid materials or ceramic–metal combinations is considered safer and less problematic [57,98,99].

In parallel, the Ceramic-on-Ceramic (CoC) combination was developed to offer extremely hard and wear-resistant surfaces with reduced particle release compared to other combinations, as shown in Figure 10. However, these materials, while advanced, present some disadvantages, such as fragility and fracture risk, and the acoustic phenomenon known as “squeaking” [32,47,49,97]. Composite ceramics, ZTA, and innovative materials like Alumina-Matrix Ceramics (AMCs) offer significant improvements in fracture and wear resistance, making them particularly suitable for young and active patients [60,82,100]. Additionally, the use of large-diameter ceramic heads has been shown to improve implant stability and reduce the risk of dislocation.

The Oxidized Zirconium-on-Polyethylene (OxZr-on-HXLPE) combination represents one of the most recent technologies, combining the high scratch and wear resistance of ZrO_2_ with the durability of HXLPE. While promising, this technology requires further clinical validation before being adopted as a standard. Innovations and advanced materials, such as new coatings and alloys developed to further improve wear resistance and reduce particle release risk, represent the future of hip prosthesis design, with increasing attention to implant customization and adaptability to the specific needs of patients [56,60].

This focus on the materials used for the femoral head and acetabular insert is justified by their direct influence on the tribological performance of the implant, while the material of the acetabular cup, although essential for structural support, has a lesser impact on overall tribology [82].

The search for new design methodologies and new materials for the creation of increasingly high-performance prostheses has never stopped over time, and since the dawn of the first applications, it has progressed and continues to progress without stopping. The use of several biocompatible materials has been tested, such as polymers (from natural to synthetic), ceramics (from glass to advanced ceramics), different types of metal alloys, and, more recently, the FGMs and several types of composite materials, trying to achieve the optimal combination in terms of biocompatibility, static and dynamic mechanical resistance, fatigue resistance, wear resistance, and resistance to chemical attacks.

**Table 7:** Comparison of the main bearing surface properties for THA applications.

Material	Chemical Reactivity	Wear Resistance	Biocompatibility	Debrits Release Risk	Survival Rates (10 Years)	Cost
MoP	Medium	Medium	Medium	High	>90%	Low
CoP	Low	Medium-High	High	Medium	>99%	Medium
MoM	High	High	Variable	Very High	80−90%	Low
CoC	Low	Very High	High	Low	>95%	High

## 10. Conclusions

THA represents a landmark achievement in modern orthopedic surgery, enabled by continuous advancements in biomaterials. The evolution from early natural materials to cutting-edge solutions, including titanium alloys (Ti), reinforced ceramics, and high-performance polymers, has profoundly enhanced the performance and durability of implants. Metallic alloys, such as stainless steel (SS) and cobalt-chrome (Co-Cr), provided the initial foundation for durable implants, while ceramics like alumina (Al_2_O_3_) and zirconia (ZrO_2_) addressed wear issues and improved biocompatibility. Concurrently, polymeric materials, such as HXLPE, have effectively mitigated problems like osteolysis and wear particle generation.

Looking ahead, several promising directions for future development emerge. One key focus is the optimization of bioactive coatings, such as Hydroxyapatite (HAP) and Bioglass, to improve osseointegration and reduce implant annual revision rates. The integration of nanotechnology offers opportunities to enhance wear resistance and mechanical properties while minimizing adverse biological reactions. Additionally, the development of advanced composites, such as metal–ceramic hybrids and antioxidant-infused polymers, holds great potential for addressing the dual challenges of wear and long-term stability.

Personalized medicine also plays a critical role in the future of THA. The ability to design patient-specific implants using additive manufacturing and advanced modeling techniques could ensure a better fit, enhanced biomechanical performance, and improved patient outcomes. Furthermore, ongoing research into reducing manufacturing costs could make these advanced materials more accessible globally, expanding the reach of high-quality hip replacements to underserved populations.

By focusing on these innovative strategies, THA has the potential to achieve unprecedented levels of reliability and effectiveness, ultimately transforming the quality of life for patients worldwide. It is important to note that this paper represents Part 1 of a broader study. While the present work furnishes a comparative analysis of biomaterials, including their clinical performance, complication rates, and revision outcomes, Part 2 focuses on the criteria and methods for selecting materials in THA. Readers interested in selection criteria regarding materials for THA application are encouraged to refer to the second part for a complete overview.

## Figures and Tables

**Table 3:** Summary of the mechanical properties and clinical metrics of ceramic materials employed for THA.

Material	Elastic Modulus [GPa]	Mechanical Strength [MPa]	Chemical Reactivity	Wear Resistance	Biocompatibility	Brittleness	Cost
Alumina	300–370	210–220	Low	Medium	High	High	Low
Zirconia	200–220	900–1250	Low	Medium	High	High	Medium
ZTA	310–360	340–350	Medium	High	High	Very High	High
Y-TZP	200–220	900–1250	Medium	High	High	Very High	Very High
HAP	80–110	38–100	High	Low	Very High	Very High	Low
Bioglass and Glass-ceramic	35–50	45–100	High	Low	Very High	Very High	Medium

**Table 5:** Summary of the application field for the main material employed for THA prosthesis fabrication (Y = yes, N = No).

Materials	Stem	Femoral Head	Liner	Acetabular Cup
Stainless Steel	Y	N	N	Y
Co-Cr Alloys	Y	Y	N	Y
Ti Alloys	Y	N	N	Y
Oxidized Zirconium	N	Y	Trial	N
Ta Alloys	Y	N	N	Y
Nb Alloys	Y	N	N	Trial
SMA	Trial	N	N	N
UHMWPE	N	N	Y	Y
HXLPE	N	N	Y	Y
PU	N	N	Trial	N
PEEK	Trial	N	Trial	Trial
Hylamer	N	N	Y	N
Alumina	N	Y	Y	N
Zirconia	N	Y	Y	N
ZTA	N	Y	Y	N
Y-TZP	N	Y	Y	N
Hydroxyapatite	Coating	N	N	Coating
Bioglass/Glass-ceramics	N	N	Trial	Trial
Carbon-Fiber-Reinforced Polymers	Y	N	Trial	Trial
Glass Fiber-Reinforced Polymers	Y	N	Trial	Trial
PEEK Composites	Y	N	Y	Y
UHMWPE + CNTs	N	N	Y	Y
HA-Coated Composites	Y	N	N	Y
Ceramic Matrix Composites	N	Y	Y	N
Metal Matrix Composites	Y	Y	N	Y
Functionally Graded Materials	Y	Trial	Trial	Y

**Table 6:** Summary of the main properties and clinical metrics of cements employed for THA.

Material	Bonding Speed	Implant Stability	Biocompatibility	Exotermic Reaction Risk	Cost
PMMA	Medium	Medium	High	High	Low
Antibiotic-Loaded Cement	Medium	Medium	High	High	High
Low-Viscosity Cement	High	High	High	Very High	Medium
Medium and High-Viscosity Cement	Medium	Very High	High	Medium	High
Beneloc	High	Very High	Very High	Low	Very High
Radiopaque-Enhanced Cement	Medium	Medium	High	Medium	Medium

## Data Availability

Not applicable.

## Supplementary Materials

The following supporting information can be downloaded at https://www.mdpi.com/article/10.3390/jfb16050179/s1. Table S1: Summary of the main metallic materials employed for THA with their benefits/drawbacks; Table S2: Summary of the main polymeric materials employed for THA with their bene-fits/drawbacks; Table S3: Summary of the main ceramic materials employed for THA with their benefits/drawbacks; Table S4: Summary of the main composite materials employed for THA with their bene-fits/drawbacks; Table S5: Summary of the main composite materials employed for THA with their bene-fits/drawbacks; Table S6: Summary of the main composite materials employed for THA with their bene-fits/drawbacks. Tables S1–S6 (References [9,10,11,12,13,14,15,16,17,18,19,20,21,22,23,24,25,26,27,28,29,30,31,32,33,34,35,36,37,38,39,40,41,42,43,44,45,46,47,48,49,50,51,52,53,54,55,56,57,58,59,60,61,62,63,64,65,66,67,68,69,70,71,72,73,74,75,76,77,78,79,80,81,82,83,84,85,86,87,88,89,90,91,92,93,94,95,96,97,98,99,100] are cited in the Supplementary Materials: Tables S1–S6).

## Abbreviations

The following abbreviations are used in this manuscript:

Al_2_O_3_	Alumina (Aluminum Oxide);
AMC	Alumina-Matrix Ceramic;
CFR-PEEK	Carbon-Fiber-Reinforced Polyetheretherketone;
CFRP	Carbon-Fiber-Reinforced Polymer;
CNT	Carbon Nanotube;
Co-Cr	Cobalt-Chrome alloy;
CoC	Ceramic-on-Ceramic;
CoP	Ceramic-on-Polyethylene;
DLC	Diamond-Like Carbon;
FGM	Functionally Graded Material;
FRCC	Fiber-Reinforced Ceramic Composite;
GFRP	Glass Fiber-Reinforced Polymer;
HAP	Hydroxyapatite;
HIP	Hot Isostatic Pressing;
HXLPE	Highly cross-linked polyethylene;
LFIT	Implied as a brand of ion-implanted Co-Cr heads;
LTD	Low-Temperature Deterioration;
MMC	Metal Matrix Composites;
MoM	Metal-on-Metal;
MoP	Metal-on-Polyethylene;
Nb	Niobium alloys;
NiTi	Nickel–titanium alloy;
OxZr	Oxidized Zirconium;
PEEK	Polyetheretherketone;
PMMA	Polymethylmethacrylate;
PMPC	Poly(2-methacryloyloxyethyl phosphorylcholine);
PTFE	Polytetrafluoroethylene;
PU	Polyurethane;
SMA	Shape Memory Alloy;
SS	Stainless steel;
Ta	Tantalum alloy;
THA	Total hip arthroplasty;
THR	Total hip replacement;
Ti	Titanium alloys;
Ti-6Al-4V	Titanium alloy composed of 6% aluminum and 4% vanadium;
Ti-13Nb-13Zr	Titanium alloy composed of 13% niobium and 13% zirconium;
TiN	Titanium Nitride;
UHMWPE	Ultra-High-Molecular-Weight Polyethylene;
Y-TZP	Yttria-Stabilized Tetragonal Zirconia Polycrystal;
ZTA	Zirconia-Toughened Alumina;
Zr	Zirconium;
ZrO_2_	Zirconia (zirconium dioxide).
