# Supplementary material for: A Comprehensive Literature Review of Total Hip Arthroplasty (THA): Part 1—Biomaterials
Source: J Funct Biomater. 2025 May 14;16(5):179. doi: 10.3390/jfb16050179 (PMC12112016; doi:10.3390/jfb16050179)
Supplement: Supplementary file 1 [file jfb-16-00179-s001.zip › jfb-3612830-supplementary.pdf]

Supplementary Data

A Comprehensive Review for Total Hip Arthroplasty (THA): Part 1 – Biomaterials

Chiara Morano, Salvatore Garofalo, Paolo Bertuccio, Agata Sposato, Irene Zappone and Leonardo Pagnotta*

Department of Mechanical, Energy and Management Engineering, University of Calabria, Via P. Bucci 44C, 87036 Rende, Italy
* Correspondence: leonardo.pagnotta@unical.it

Table S1. Summary of the main metallic materials employed for THA with their benefits/drawbacks.

Material	Benefits	Drawbacks	Applications	References
Stainless Steel (SS)	High mechanical strength and corrosion resistance; relatively inexpensive; good biocompatibility.	Corrosion over time; Releasing metal ions with risk of metallosis and bone loss; stress shielding due to high elastic modulus.	Femoral stems and temporary implants; low-cost applications; elderly or less active patients.	[9,10,12–14,23,24,26–31]
Cobalt-Chrome Alloys (Co-Cr)	Excellent wear and corrosion resistance; high mechanical strength; high loads resistance.	Releasing metal ions that can cause adverse reactions; problematic for patients sensitive to metals.	Femoral heads and articulating surfaces; Young and active patients; Revision prostheses for its robustness.	[9,14,19,20,22,26–29,31–34]
Titanium Alloys (Ti)	Excellent biocompatibility; low density, corrosion resistance; elastic modulus similar to human bone;	Lower wear resistance; requires advanced surface treatments to enhance durability; risk of galvanic corrosion when combined with other metallic alloys.	Femoral stems and acetabular cups; Young and active patients; well suitable in cementless configurations.	[9,10,14,24,26,35–38]
Oxidized Zirconium (OxZr)	Superior wear resistance reduces liner wear osteolysis; excellent biocompatibility and low friction coefficient.	High costs and complex manufacturing processes; limited data about long-term performance.	Femoral heads for THA, particularly in younger patients or those with metal sensitivities.	[10,17,19,24,39–41]
Tantalum Alloys (Ta)	Excellent osseointegration due to its porous structure; high implant stability and prosthesis longevity.	High cost and rigidity; optimal positioning is challenging; rigidity can cause stress on surrounding tissues.	Production of acetabular cups and femoral stems; Revision surgeries and in patients with compromised bone.	[37,42–46]
Niobium Alloys (Nb)	Excellent corrosion resistance, high biocompatibility, and hypoallergenic properties.	High manufacturing costs; relatively limited long-term clinical data.	Femoral stems and acetabular cups; application in cases requiring high corrosion resistance and mechanical durability.	[9,19,20,47–50]
Shape Memory Alloys (SMA)	Adaptability to natural movements; low risk of stress shielding; high biocompatibility,	Release of metallic ions (Nickel) that can cause adverse reactions; potential long-term biocompatibility issues.	Femoral stems, fixation plates, and modular actuator systems;	[18,22,27,51–53]

	and corrosion resistance.			
Advanced Metallic Materials	High biocompatibility and mechanical strength; coatings enhance corrosion and wear resistance; low risk of metal ion release.	More expensive and complex to produce; limited long-term clinical data on their use.	Femoral stems, acetabular cups, and articulating surfaces in THA, enhancing durability and reducing complications related to traditional metallic materials.	[9–12,25,26]

Table S2. Summary of the main polymeric materials employed for THA with their benefits/drawbacks.

Material	Benefits	Drawbacks	Applications	References
Ultra-High-Molecular-Weight Polyethylene (UHMWPE)	Excellent wear resistance; high biocompatibility.	Susceptible to oxidation; wear debris formation; low implant's longevity in certain applications.	Acetabular components.	[9–11,13,17,19,23,54–56]
Highly Cross-linked Polyethylene (HXLPE)	High wear resistance; reduced wear particle production; high implant life; improved prosthesis stability.	Low hardness and rigidity; free radicals production; oxidation during storage; fatigue fractures and oxidation issues.	Acetabular liners in hip prosthes, expecially for larger diameter femoral heads.	[10,55,57,58]
Polyurethane (PU)	Excellent elasticity and flexibility; reduced stress concentrations; customizable for various implant designs.	Potential degradation over time; low biocompatibility and risk of adverse tissue reactions.	Acetabular liners and other components where elasticity and wear resistance are valuable.	[59–62]
Polyetheretherketone (PEEK)	Modulus of elasticity similar to bone; Radiolucency; Useful for composites fabrication.	Low wear resistance; high production costs.	Acetabular liners and femoral stems; adaptable for other orthopedic applications, such as spinal implants and cranial plates.	[17,18,22,56,63–66]
Polytetrafluoroethylen (PTFE)	Low coefficient of friction; excellent chemical resistance.	Poor wear resistance; production of wear particles.	Surfaces of hip prostheses but replaced by other materials; no longer used in THA.	[10,12,17,56,67]
Polyamide (Nylon)	Strong mechanical properties (high	Moisture absorption,	Acetabular liners and bearing	[68–70]

	tensile strength and good elasticity); initial wear resistance and toughness.	swelling, and softening; insufficient wear resistance under high-stress conditions; wear particle production.	surfaces in hip prostheses; replaced by more advanced materials; no longer used.	
Hylamer (Enhanced UHMWPE)	Improved initial wear resistance; reduced wear debris; enhanced implant longevity.	Oxidative degradation; increased wear rates under fatigue conditions; higher failure rates.	Acetabular liners and other bearing surfaces in hip prostheses; replaced by more advanced materials; no longer used.	[18,19,64,71]
Advanced and Emerging Polymers	Enhanced wear resistance, reduced oxidative degradation, and potential improvements in implant longevity.	Limited long-term clinical data; antioxidant integration, needs further validation in real-world settings; high cost.	Acetabular liners, dual mobility systems, and monoblock cementless components; Applications may expand as more data become available.	[18,19,72,73]

Table S3. Summary of the main ceramic materials employed for THA with their benefits/drawbacks.

**Material**	**Benefits**	**Drawbacks**	**Applications**	**References**
Alumina (Al_2O_3)	High hardness and wear resistance; biocompatibility and low chemical reactivity, low osteolysis risk.	Intrinsic brittleness; need to guarantee high material purity.	Femoral heads and acetabular inserts; suitable for young, active patients.	[9–11,17,18,21,74,76–78]
Zirconia (ZrO_2)	High fracture resistance and toughness; excellent biocompatibility and low friction; low osteolysis risk.	Phase transformation can reduce toughness, increasing wear; brittleness.	Femoral heads and acetabular components; paired with reticulated polyethylene inserts or composite materials.	[9,10,17–19,21–23,25,56]
Zirconia Toughened Alumina (ZTA)	High toughness that prevents crack propagation; high biocompatibility.	Complex and costly production process; it requires careful handling to avoid fractures.	Femoral heads and acetabular inserts; adapt for young, active patients.	[11,17,21,22,56,67]
Yttria-Stabilized Tetragonal Zirconia Polycrystals	High fracture resistance and toughness; excellent biocompatibility and smooth surface; low friction and wear.	Risk of degradation at low temperatures (aging); fragility	Femoral heads and acetabular components; suitable for young and very active patients.	[9,17,22,56]

(Y-TZP)		requires careful handling.		
Hydroxyapatite (HAP)	Excellent biocompatibility and osteoconductive properties; promote bone growth and integration with implants.	Inherently brittle with low tensile and compressive strength; unsuitable as a standalone material.	Used as a coating for metallic implants to enhance osseointegration; explored for composite materials and scaffolds for bone regeneration.	[13,18,19,61]
Bioglass and Glass-ceramics	Excellent mechanical and thermal properties; promotes osteointegration and bone regeneration.	Brittle and low mechanical strength; require careful handling to prevent fracture.	Coating for implants to enhance osteointegration and as a bone graft substitute.	[75,79–81]

Table S4. Summary of the main composite materials employed for THA with their benefits/drawbacks.

Material	Benefits	Drawbacks	Applications	References
Carbon Fiber-Reinforced Polymers (CFRPs)	High strength-to-weight ratio; Good fatigue resistance; Reduced stress shielding due to similar elasticity to bone.	Potential for wear debris generation; Higher cost compared to metals.	Used in femoral stems to reduce stress shielding and improve load transfer to the bone.	[9,25,64,66,84]
Glass Fiber-Reinforced Polymers (GFRPs)	Lightweight; Good corrosion resistance; Easier to manufacture in complex shapes.	Lower mechanical strength compared to CFRPs; Susceptibility to moisture absorption.	Applied in acetabular cups and other non-load bearing components.	[9,25,66,84]
Polyetheretherketone(PEEK) Composites	Biocompatible; Low wear rates; Similar modulus of elasticity to bone, reducing stress shielding.	Limited clinical history compared to other materials; High cost	Used in femoral components and intervertebral implants, with ongoing research for broader orthopedic applications.	[9,25,64,66]
UHMWPE Reinforced with CNTs	Improved wear resistance; Enhanced mechanical properties compared to standard UHMWPE.	Complexity in Manufacturing; Potential health risks associated with carbon nanotube exposure.	Utilized in bearing surfaces to extend the life of implants and reduce revision rates.	[9,84]
Hydroxyapatite	Promotes bone in-growth;	Potential for delamination of	Applied as a coating on metallic implants	[85]

(HAP) Coated Composites	Biocompatible; Improves osteointegration.	the coating; Limited load-bearing capacity.	and as a filler in composite materials to enhance bone integration.	
Ceramic Matrix Composites (CMCs)	High wear resistance; Low friction; Biocompatible.	Brittle nature; Higher manufacturing costs.	Used in bearing surfaces and femoral heads to reduce wear and improve the longevity of implants.	[23,56]
Hybrid Composites	Combines properties of different materials for optimized performance; Tailored mechanical properties.	Complexity in design and manufacturing; Potential for interface issues between materials.	Used in femoral stems and acetabular components for a balance of strength, flexibility, and biocompatibility.	[19,100]
Metal Matrix Composites (MMCs)	High strength and wear resistance; Excellent load-bearing capacity.	Potential for corrosion at the interface; Higher density compared to polymer composites.	Applied in load-bearing components like femoral heads and stems to enhance durability and wear resistance.	[23]
Graded Materials	Tailored mechanical properties that mimic the gradient of natural bone; Improved stress distribution.	Complexity in manufacturing; Higher production costs.	Used in femoral stems and acetabular components to optimize the interface between implant and bone.	[87,88]

Table S5. Summary of the main composite materials employed for THA with their benefits/drawbacks.

Material	Benefits	Drawbacks	Applications	References
Polymethylmethacrylate (PMMA)	Immediate fixation; Good mechanical properties; Ease of use.	Exothermic reaction can cause thermal necrosis; Potential for long-term loosening.	Standard for prosthesis fixation in THA, especially in patients with poor bone quality.	[89,91,95]
Antibiotic-Loaded Cement	Reduces risk of postoperative infections; Particularly useful in revision surgeries.	Potential development of antibiotic resistance; Increased cost.	Primarily used in revision surgeries and patients at high risk of infection.	[89–91,95,96]
Low-Viscosity Cement	Better penetration into cancellous	More complex handling;	Indicated for cases with weak or osteoporotic	[90,95]

	bone; Increased implant stability.	Higher technical requirements.	bone, where deeper penetration is needed.	
Medium and High-Viscosity Cement	Ease of handling; Fast setting times.	Risk of poor penetration if not applied correctly.	Used in standard conditions where ease of handling is essential for successful surgery.	[95]
Low-Exothermic Cement (Boneloc)	Reduces heat generated during polymerization; Minimizes risk of thermal necrosis.	Inferior mechanical properties compared to conventional PMMA cements.	Used in contexts where temperature control is critical, though less common due to inferior mechanical properties.	[95]
Radiopaque-Enhanced Cement	Improved radiographic visualization; Facilitates postoperative monitoring.	Possible reduction in mechanical strength due to additives.	Used to ensure clear radiographic visibility of the implant post-operation.	[89,91]

Table S6. Summary of the main composite materials employed for THA with their benefits/drawbacks.

Material	Benefits	Drawbacks	Used materials	Future materials	Applications	References
Metal-on-Polyethylene (MoP)	Easy handling, low cost, extensive clinical experience, reduced wear with HXLPE	Risk of oxidation and mechanical degradation of polyethylene over time	*Femoral head*: Metal (Co-Cr) *Insert*: Polyethylene, HXLPE	PEEK reinforced with carbon fibers for the insert, addition of antioxidants (Vitamin E, MWCNT)	Widely used in elderly patients, growing interest in using stabilized HXLPE in younger patients	[32,47,97]
Ceramic-on-Polyethylene (CoP)	Greater wear resistance than MoP, suitable for patients allergic to metals	Risk of ceramic head fracture, higher cost	*Femoral head*: Ceramic (Al_2O_3, ZrO_2, OxZr) *Insert*: Polyethylene, HXLPE	Advanced ceramics (Biolox Delta), modified zirconium oxide (Oxinium) for the femoral head	Preferred for young and active patients, popular in Central Europe	[56,60,82]
Metal-on-Metal (MoM)	Reduced mechanical wear, better	Release of metal ions, adverse	*Femoral head and insert*:	Advanced coatings (TiN, TiNbN) for	Strongly reduced use due to	[32,49,98]

	implant stability	reactions in soft tissues	Metal (Co-Cr)	both components	complications related to metal ion release	
Ceramic-on-Ceramic (CoC)	Excellent wear resistance, high biocompatibility	Fragility, risk of squeaking and fractures, high cost	*Femoral head and insert*: Ceramic (Al_2O_3, ZrO_2, Biolox Delta)	Advanced ceramic composites (Zirconia-toughened alumina) for both components	Popular in young and active patients, limited use due to high cost and risk of complications	[32,47,49,82,97,100]
Oxidized Zirconium-on-Polyethylene (OxZr-on-HXLPE)	High scratch and wear resistance, biocompatibility	Relatively new technology, need for further long-term studies	*Femoral head*: Zirconium Oxide (Oxinium) *Insert*: Polyethylene, HXLPE	New coatings and alloys to improve wear resistance of the femoral head	Promising for young patients, currently under evaluation for broader use	[56,60]
